# Entropy *vs.* Energy Waveform Processing: A Comparison Based on the Heat Equation

**DOI:** 10.3390/e17063518

**Published:** 2015-05-25

**Authors:** Michael S. Hughes, John E. McCarthy, Paul J. Bruillard, Jon N. Marsh, Samuel A. Wickline

**Affiliations:** 1 Pacific Northwest National Laboratory, 902 Battelle Blvd., Richland, WA 99354, USA; 2 Department of Mathematics, Washington University in St. Louis, 1 Brookings Dr., St Louis, MO 63130, USA; 3 School of Medicine, Washington University in St. Louis, 660 S. Euclid Ave, St Louis, MO 63110, USA

**Keywords:** information wave, optimal detection, entropy image, joint entropy

## Abstract

Virtually all modern imaging devices collect electromagnetic or acoustic waves and use the energy carried by these waves to determine pixel values to create what is basically an “energy” picture. However, waves also carry “information”, as quantified by some form of entropy, and this may also be used to produce an “information” image. Numerous published studies have demonstrated the advantages of entropy, or “information imaging”, over conventional methods. The most sensitive information measure appears to be the joint entropy of the collected wave and a reference signal. The sensitivity of repeated experimental observations of a slowly-changing quantity may be defined as the mean variation (*i.e.*, observed change) divided by mean variance (*i.e.*, noise). Wiener integration permits computation of the required mean values and variances as solutions to the heat equation, permitting estimation of their relative magnitudes. There always exists a reference, such that joint entropy has larger variation and smaller variance than the corresponding quantities for signal energy, matching observations of several studies. Moreover, a general prescription for finding an “optimal” reference for the joint entropy emerges, which also has been validated in several studies.

## 1. Introduction

All applications of imaging technology begin with collection of a flux, either electromagnetic [1] or acoustic [2], and nearly all images produced by current technology, e.g., for remote sensing or medical imaging, are representations based on some type of scattered energy. The reasons for this are mainly historical, since the detectors are typically developed by physicists and electrical engineers who are intimately acquainted with the wave equation and transmission line theory where conservation of energy is a central concept. On a more practical level, transduction elements are frequently characterized in terms of energy conversion efficiencies, making it natural to think of the subsequent image formation process in terms of either electromagnetic or acoustic field energy. From this perspective, if the received energy arriving during some small interval of time must be reduced to a single number in order to compute an image pixel value, energy is the obvious choice.

For instance, in the field of medical ultrasonics, tumor detection and tissue classification with ultrasound remain a highly useful and clinically relevant approach (liver, kidney, prostate, breast, heart, eye, thyroid, pancreas, gall bladder, *etc.*). A number of teams, including those of Insana [3–5], Forsberg [6,7], Deng [8], and others, have pursued novel data acquisition and reduction schemes (spectral, cepstral, wavelet, elastographic, harmonic, *etc.*) to augment diagnostic power from traditional radio frequency data, and progress continues apace. Nevertheless, although hardware improvements in clinical imaging systems over the last 50 years have dramatically improved the ability of ultrasound to display tissue features, resident signal processing algorithms have not evolved much beyond fundamental presentation of the energy of backscattered compressional waves.

Since all detectors have a finite response time, their output is essentially an integral of the incoming flux that they measure taken over a very short time interval. As such, it is natural to ask if other integrals or functionals of the incident flux might also have utility if represented as images [9–14].

In fact, numerous experimental studies have demonstrated the utility of information theoretic quantities for this type of analysis of experimentally-measured waveforms. In the standard application of information theory, as initiated by Shannon, the random variables (*i.e.*, waveforms, which we will denote *f* and *g* defined on [0, 1]) are assumed to have the same underlying distribution [15]. However, additional assumptions, such as differentiability, permit computation of distributions, both individual and joint, directly from the measured waveforms. Operationally, the ability to differentiate experimentally-measured waveforms, which contain noise, requires regularization. For our studies, this is accomplished using optimal smoothing splines [16]. Differentiability combined with regularization then permits computation of the distributions for individual waveforms, or pairs of waveforms in the case of joint distributions. Information-theoretic quantities may then be computed from these distributions in an approach that corresponds more closely to that initiated by Kolmogorov and Chaitan, where entropy is a measure of the intrinsic complexity of individual mathematical objects [17,18]. The underlying assumption in experimental applications of these quantities is that the waveforms, usually acquired in scattering measurements, faithfully capture the complexity of the interrogated scattering architectures. The merit of this strategy has been demonstrated in several experimental studies, which have investigated the sensitivities of several entropies for the detection of small changes in waveforms acquired in acoustic experiments [19–24].

To date, the most sensitive of these is a joint entropy of two waveforms, one acquired in an acoustic backscatter (*i.e.*, an echo) measurement from an experimental specimen, *f*(*t*), and the other a reference, *g*(*t*), which may be obtained separately by experiment or by theoretical modeling [25]. To compute this entropy for differentiable waveforms *f*(*t*), *g*(*t*), we must calculate their joint distribution *w_f,g_*, which is not a function, but a tempered distribution [25]. Thus, calculations based on *w_f,g_* require “coarse-graining” on a uniform grid of *C* × *C* squares covering the *x, y*-plane to obtain a discrete joint probability distribution *p^C^*(*j, k*) from *w_f,g_* by integrating its product with smoothed versions of the characteristic functions of these squares (Equations 40 to 44 of [25]). This is followed by a limiting process where the grid size, *C*, is taken to zero. For instance, the calculation of the joint entropy begins with the following computation:

(1) $$H_{f,g}=\underset{C\to 0}{\text{lim}}H^{C}(f,g)=\underset{C\to 0}{\text{lim}}\sum_{j,k}p^{C}(j,k)\text{ log }[p^{C}(j,k)]$$

In the limit where *C* → 0 and *f*, *g* are piecewise differentiable functions on [0,1] (without any intervals of constancy), Equation (1) becomes [25]:

(2) $$H_{f,g}=-\frac{1}{2}\int_{0}^{1}\frac{\text{min}[|f\prime (t)|,|g\prime (t)|]}{\text{max}[|f\prime (t)|,|g\prime (t)|]}dt-\int_{0}^{1}\text{log }[\text{max}[|f\prime (t)|,|g\prime (t)|]]dt$$

In this note we determine conditions on *g*(*t*) that maximize the sensitivity of *H_f,g_* to small changes in *f*(*t*). This requires a lengthy calculation.

We will be comparing this sensitivity to that of the signal energy, *E_f_*, of *f*(*t*) defined by:

(3) $$E_{f}=\int_{0}^{1}f{\left(t\right)}^{2}dt$$

Numerous studies have shown that entropy signal receivers are always at least as sensitive as *E_f_* is to small changes in *f* (see Equation (5)). In fact, in many cases, it is actually much more sensitive [19–25].

**Figure 1:**
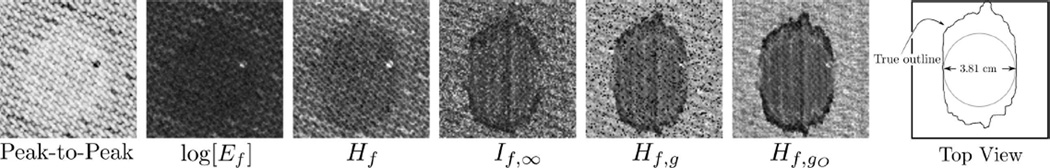
Materials characterization using entropy signal receivers. The derivation of the prescription for the reference *g* that permits the improvement in contrast between the *H_f,g_* and *H_f,gO_* images is the subject of this study. (**Far left**) Sample diagram showing intended circular defect shape and the actual defect shape as revealed by several entropy images and verified by eventual destructive examination.

A typical result for materials characterization is shown in Figure 1 [26], which shows images of a graphite/epoxy composite laminate scanned using a 2.5-MHz transducer on a 101 × 101 point grid. The backscattered ultrasound was digitized for off-line analysis. The peak-to-peak image was produced using the peak-to-peak amplitudes of the waveforms. The rest were produced using a moving window (128 points long) analysis to produce a stack of images corresponding to different depths whose minima were then projected onto a single image-plane to permit rapid analysis of the entire image set. This projection scheme is frequently used to reduce the amount of data that must be inspected or in the case where the defect is not confined to a narrow range of depths. The *E_f_* image was produced using Equation (3). The *H_f_* [20], *I*_*f*, ∞_ [27], *H_f,g_* ([25] or Equation (2)) and *H_f,gO_* are entropy images.

Similar results have also been obtained in medical imaging [27–38].

## 2. The Main Result

All of our studies have been based on the (fairly typical) situation where an experimentalist acquires waveforms of a few microseconds duration over a much greater period of time spanning minutes or longer. Thus, there are two time scales: the long experimental time scale and a much shorter measurement time scale (which we have parametrized on the interval [0, 1], the domain of *f*(*t*) and *g*(*t*)). If we denote a measurement at time *t* by M_*t*_, then the sensitivity is defined by [39]:

(4) $$c≔\frac{\text{mean}(\{M_{i}-M_{0}:i=1,\ldots ,n\})}{\text{standard error}(\{\Delta_{i}:i=1,\ldots ,n\})}$$

It is essentially the noise-normalized change in a physical quantity at different measurement times. The situation also describes measurements where data (*i.e.*, *f*(*t*), *g*(*t*)) are acquired at different times between which the measurement device has been moved; for instance, Figure 1, where the experimental times map to different spatial locations in the experimental specimen.

For evaluation of receiver sensitivities in theoretical studies, we will maximize the similar quantity:

(5) $$\frac{\langle \delta H_{f+\sigma \varsigma ,g}[\eta ]\rangle }{\sqrt{\text{Var}[\delta H_{f+\sigma \varsigma ,g}[\eta ]]}}$$

where the product σς(*t*) captures the impact of Gaussian noise on the experimental measurement (ς(*t*) is a functional of a Brownian path (see Equation (15)) and σ is a scalar representing the signal-to-noise ratio). On the other hand, the perturbing function εη(*t*), where ε is small and η ∈ *C*^1^[0, 1], models the effect of the variation of the scattering architecture, as captured in Figure 1. Together, these account for the specific form of the measured function, which we write as (*f* + εη +σς) (*t*); all of these functions will be discussed fully in the next section. The experimental change referred to prior to Equation (4) is then quantified mathematically by the directional derivative:

(6) $$\delta H_{f+\sigma \varsigma ,g}[\eta ]≔{\frac{dH_{f+\varepsilon \eta +\sigma \varsigma ,g}}{d\varepsilon }|}_{\varepsilon =0}$$

The measure of noise is:

(7) $$\text{Var}[\delta H_{f+\sigma \varsigma ,g}[\eta ]]≔\langle {\left(\delta H_{f+\sigma \varsigma ,g}[\eta ]\right)}^{2}\rangle -{\langle \delta H_{f+\sigma \varsigma ,g}[\eta ]\rangle }^{2}$$

where all of the means are computed as Wiener integrals as discussed below. Similar definitions hold for the signal energy *E_f_*.

After carefully-defining the quantities, *f*(*t*), η(*t*), σ, ς(*t*), in terms of a physical measurement model, having finite time resolution width Δ ≪ 1, we calculate the variations δ*H*_*f*+σς,*g*_ [η] and δ*E*_*f*+σς_[η] in Section 4. The mean values of these are then calculated in Section 5 as Wiener integrals. The joint entropy is considered first in Section 4.1. After deriving the mean variation as a Wiener integral, we rewrite it in Section 5.1.1 as a Lebesgue integral. This integral represents a family of solutions to the heat equation with initial conditions defined on the real line. However, the noise-level plays the role usually held by time.

The results of the calculations may be summarized as:

### Theorem 1

*Suppose that f is in C*^3^[0, 1] *and there exists t*_0_ ∈ (0, 1), *such that f*′(*t*_0_) = 0 *and f*″(*t*_0_) ≠ 0. *Then, there exists C*_1_ > 0, *such that for all C*_2_ > 0, *there exists a piecewise differentiable function g, independent of* η, *such that:*

$$\underset{\sigma \to 0}{\text{lim}}|\langle \delta H_{f+\sigma \varsigma ,g}[\eta ]\rangle |\geq C_{2}|\eta \prime (t_{0})|-C_{1}{\left\|\eta \prime \right\|}_{\infty }$$

*In particular, provided* η′(*t*_0_) ≠, *one can make* $\underset{\sigma \to 0}{\text{lim}}\langle \delta H_{f+\sigma \varsigma ,g}[\eta ]\rangle$ *arbitrarily large*.

In nearly all practical situations, such a *t*_0_ exists. We prove this theorem by constructing an example reference at the end of Section 5.1.1.

There are actually many such *g*(*t*). A good way to construct examples is, roughly, to pick a zero, *t*_0_, of *f*′(*t*) and have *g*′(*t*) be a non-zero constant on one side of *t*_0_, while on the other side, it differs from *f*′(*t*) by only a small constant 1 ≫ ω > 0. In the limit as ω → 0, |δ*H*_*f*+σς,*g*_[η]| > 2|η′(*t*_0_)| log[1/ω]/*f*″(*t*_0_). Exact details are provided in the proof, where a bound on the size of *g*(*t*) is also provided.

On the other hand, the variation for the signal energy, provided by Equation (81), is calculated in Section 4.2. If *f* and η are bounded, then in the noise-free limit, it is (see Equation (51)):

(8) $$\delta E_{f+\sigma \varsigma }[\eta ]=2\int_{0}^{1}f(t)\eta (t)dt$$

which is bounded.

Thus, if η′(*t*_0_) ≠ 0, there exists *g*, such that:

(9) $$\underset{\sigma \to 0}{\text{lim}}|\langle \delta H_{f+\sigma \varsigma ,g}[\eta ]\rangle |\geq \underset{\sigma \to 0}{\text{lim}}|\langle \delta E_{f+\sigma \varsigma }[\eta ]\rangle |$$

There are many theorems describing the behavior of solutions to the heat equation for small time discussed in Section 5.1.2. These results are used in Section 6 to prove:

### Theorem 2

*Suppose K*_0_ > 0 *is a constant, f*, η *are in C*^1^[0, 1] *and g is piecewise C*^1^[0, 1]. *Let* 0 < α < 1/6. *Suppose that, except at finitely many points*, |*g*′(*t*)| ≠ |*f*′(*t*)| *and* |*g*′(*t*)| ≥ *K*_0_Δ^α^.

- *Then, for* Δ *small, there exists a constant K*_1_ *that depends on* ‖η′‖_∞_, *but not on g or* σ, *such that:*
  (10) $$\text{Var}[\delta H_{f+\sigma \varsigma ,g}[\eta ]]\leq K_{1}\Delta^{1-6\alpha }\sigma^{2}+O[\Delta \sigma^{2}+\sigma^{4}]$$
- *There exists K*_2_ > 0, *that depends on* ‖η‖_∞_, *but not on* Δ *or* σ, *such that:*
  (11) $$\text{Var}[\delta E_{f+\sigma \varsigma }[\eta ]]=K_{2}\Delta^{1/2}\sigma^{2}+O[\sigma^{3}]$$

Consequently, given the freedom to choose *g*(*t*), the sensitivity of joint entropy analysis can always be made greater than that of signal energy analysis. For instance, choosing α = 1/16 and ω = Δ^1/32^ and setting *g*′(*t*) = *f*′(*t*) − ω for *t* < *t*_0_ and *g*′(*t*) = 1 for *t* > *t*_0_ produces a reference that satisfies the requirement Δ^1/32^ ~ |*g*′(*t*)| ≫ Δ^α^ ~ Δ^1/16^ and leads to δ*H*_*f*+σς,*g*_[η] ~ η′(*t*_0_) log[Δ]/(4*f*″(*t*_0_)); so that the confidence for *H_f,g_* is *O* [σ^−1^Δ^−5/16^ log[Δ]], whereas the signal energy will have confidence $O[\sqrt{\sigma }\Delta^{-1/4}]$, since δ*E*_*f*+σς_[η] = *O*[1].

## 3. Approach

### 3.1. The Physical Setup

We assume that we have an experimental system that is measured by some means, e.g., interrogated by waves, π(*t*), of some sort, such as microwave or ultrasonic radiation, and that we collect some portion of the waves scattered in this system to obtain both perturbed, *f*_pert._(*t*), and unperturbed functions, *f*_unpert._(*t*), for times normalized by the appropriate choice of units to lie between zero and one. The difference |*f*_unpert._(*t*) − *f*_pert._(*t*)| is assumed to be small and to be caused physically by a change in the system as measured at different spatial locations (as in Figure 1) and/or different times if the system is evolving. We also assume that there is Gaussian distributed noise in the system and that the noise functions for all measurements are represented by Brownian paths, which we will always denote by *x*(*t*) or, when there are multiple Brownian paths that must be distinguished, by *x_i_*(*t*). Moreover, we assume that these functions, which all have a variance of one, are scaled by an experimentally-determined signal-to-noise ratio, σ, which will typically have positive values much less than one-tenth.

If the system is linear, with initial impulse-response function Λ(*t*) and final perturbed impulse-response function Λ(*t*) + ελ(*t*), these functions may be represented mathematically as:

(12) $$f_{\text{unpert}.}(t)=\int_{0}^{1}\Lambda (t-t_{1})\pi (t_{1})dt_{1}+\sigma x_{1}(t)$$

$$f_{\text{pert}.}(t)=\int_{0}^{1}[\Lambda (t-t_{1})+\varepsilon \lambda (t-t_{1})]\pi (t_{1})dt_{1}+\sigma x_{2}(t)$$

If the system is non-linear, these equations become approximate representations of *f*_unpert._(*t*) and *f*_pert._(*t*) in the case where the perturbation to the system is small.

All experimental systems have finite time resolution. In addition, an experimentalist may deliberately signal average successive measurements to cancel noise. Both of these facts may be expressed using convolution of ideal experimental measurements, such those in Equation (12), with a finite measurement function, μ_Δ_(*t*) of limited time duration, *i.e.*, supp(μ_Δ_) ⊊ [0, 1]. This requires us to replace Equation (12) by:

(13) $$f_{\text{unpert}.}(t)=\int_{0}^{1}\mu_{\Delta }(t-t_{2})[\int_{0}^{1}\Lambda (t_{2}-t_{1})\pi (t_{1})dt_{1}+\sigma x_{1}(t_{2})]dt_{2}\;=f(t)+\sigma \varsigma (t)$$

and:

(14) $$f_{\text{pert}.}(t)=\int_{0}^{1}\mu_{\Delta }(t-t_{2})[\int_{0}^{1}[\Lambda (t_{2}-t_{1})+\varepsilon \lambda (t_{2}-t_{1})]\pi (t_{1})dt_{1}+\sigma x_{2}(t_{2})]dt_{2}$$

$$f_{\text{pert}.}(t)=f(t)+\varepsilon \eta (t)+\sigma \varsigma (t)$$

where,

$$f(t)=\int_{0}^{1}\int_{0}^{1}\mu_{\Delta }(t-t_{2})\Lambda (t_{2}-t_{1})\pi (t_{1})dt_{1}dt_{2}$$

$$\eta (t)=\int_{0}^{1}\mu_{\Delta }(t-t_{2})\int_{0}^{1}\lambda (t_{2}-t_{1})\pi (t_{1})dt_{1}dt_{2}$$

(15) $$\varsigma (t)=\int_{0}^{1}\mu_{\Delta }(t-t_{2})x_{2}(t_{2})dt_{2}=\int_{0}^{1}x_{2}(t_{2})dM_{t}(t_{2})$$

with:

(16) $$\frac{d}{dt_{2}}M_{t}(t_{2})=\mu_{\Delta }(t-t_{2})$$

Physically, *f*(*t*) is the “well-behaved”, *i.e.*, noise-free and smooth, part of the linear response of the experimental system (as described by Λ(*t*)) to the probing “waveform”, π(*t*_1_), while η(*t*) is the noise-free and smooth change in the system, resulting from the perturbation of the system, as represented by the function λ(*t*).

We will additionally assume that,

(17) $${\left\|\eta \prime \right\|}_{\infty }<\infty$$

for later use.

Later, we will also need ς′(*t*):

(18) $$\varsigma \prime (t)=\int_{0}^{1}\frac{d}{dt}\mu_{\Delta }(t-t_{2})x_{2}(t_{2})dt_{2},\;=-\int_{0}^{1}x_{2}(t_{2})dm_{t}(t_{2})$$

with:

(19) $$m_{t}(t_{2})≔\mu_{\Delta }(t-t_{2})$$

We will also assume that μ_Δ_(*t*) is non-negative; typically, a unit step function of width less than one whose derivatives we will treat in the sense of distributions. We have from Equation (14):

(20) $$f_{\text{pert}.}^{\prime }(t)=f\prime (t)+\varepsilon \eta \prime (t)+\sigma \varsigma \prime (t)$$

which we will need for the evaluation of Equation (2).

### 3.2. Characteristics of the Measurement Window μ_Δ_(t) Relevant to the Variation and Variance of H_f+εη+σς,g_

Up to this point, we have not specified the experimental window function μ_Δ_(*t*). While many different choices for this function are possible, the most common is a unit height step function of width much smaller that the total time of the experimental measurement, which we have scaled to be one. Experimental conditions usually provide for at least sixteen sample points in this window. This is motivated by the experimental goal of selecting digitizer equipment and settings so that the experimental window function is very small compared to the scale over which the input waveform exhibits significant change. Consequently, for typical measurements, we have values of ‖*m_t_*‖ ≪ 1/16. To make these comments more precise, we will define:

(21) $$\mu_{\Delta }(t)=\frac{1}{\sqrt{\Delta }}\chi_{\left(0,\Delta \right]}(t)\chi_{\left[0,1\right]}(t)$$

where χ_(0,Δ]_(*t*) is the characteristic function of (0, Δ]. This function has finite total variation and *m_t_*(1) = μ_Δ_(*t* − 1) = 0, for all *t* ∈ [0, 1], which we must always have for technical reasons (see the Appendix on Wiener integrals Equations (128) and (146)). Later, we will also need, for the calculation of the variance of joint entropy and its variation, the relation (jointly continuous in *s* and *t*):

(22) $$\langle m_{t},m_{s}\rangle =\int_{0}^{1}m_{t}(t_{2})m_{s}(t_{2})dt_{2}=\{\begin{array}{cc} 0, & \text{if}|t-s|\geq \Delta \\ \frac{\Delta -|t-s|}{\Delta }, & \text{if}|t-s|<\Delta \text{ and }\{t,s\}⊄[1-\Delta ,1]\\ \frac{1-\text{max}(s,t)}{\Delta }, & \text{if}|t-s|<\Delta \text{ and }\{t,s\}\subset [1-\Delta ,1] \end{array}$$

and using ‖*m_t_*‖ to denote the *L*^2^-norm on [0, 1]:

(23) $$\|m_{t}\|=\{\begin{array}{cc} 1, & \text{if }t\in [0,1-\Delta ]\\ \sqrt{\frac{1-t}{\Delta },} & \text{if }t\in [1-\Delta ,1] \end{array}$$

### 3.3. Characteristics of the Measurement Window μ_Δ_(t) Relevant to the Variation and Variance of E_f+εη+σς_

If we define:

(24) $$M_{t}(t_{2})=\int_{t_{2}}^{1}\mu_{\Delta }(t-s)ds=\{\begin{array}{cc} \sqrt{\Delta }, & \text{if }t_{2}<t-\Delta \\ -\frac{1}{\sqrt{\Delta }}t_{2}+\frac{1}{\sqrt{\Delta }}t, & \text{if }t-\Delta <t_{2}<t\\ 0, & \text{if }t<t_{2} \end{array}$$

and:

(25) $$\|M_{t}\|=\sqrt{t\Delta -\frac{2}{3}\Delta^{2}}$$

Then, the last term in Equation (13) may be written as:

(26) $$\varsigma (t)=\int_{0}^{1}\mu_{\Delta }(t-t_{2})x_{1}(t_{2})dt_{2}=\int_{0}^{1}x_{1}(t_{2})dM_{t}(t_{2})$$

We also note for later use:

(27) $$\langle M_{t_{1}},M_{t_{2}}\rangle =\int_{0}^{1}M_{t_{1}}(s)M_{t_{2}}(s)ds=\text{min}(t_{1},t_{2})\Delta +O[\Delta^{2}]$$

In particular, if 0 ≤ *t*_1_, *t*_2_ ≤ 1, then we have the order relation:

(28) $$\frac{\langle M_{t_{1}},M_{t_{2}}\rangle }{\left\|M_{t_{1}}\right\|}=\frac{\text{min }(t_{1},t_{2})}{\sqrt{t_{1}}}\sqrt{\Delta }+O[\Delta^{2}]$$

## 4. Calculation of the Variations of Joint Entropy and Signal Energy

### 4.1. Calculation of the Variation, δH_f+σς,g_(η)

We now calculate the average change in Equation (2) when *f*(*t*) is perturbed by the function εη(*t*) + σς(*t*) as shown in Equation (20). The calculation is broken into two parts.

#### 4.1.1. The First Term

To begin with, we will focus on the first term in Equation (2). Using the terms defined above this is:

(29) $$-\frac{1}{2}[\int_{0}^{1}\frac{\left|f\prime (t)+\varepsilon \eta \prime (t)+\sigma \varsigma \prime (t)|+|g\prime (t)\right|}{\text{max}[|f\prime (t)+\varepsilon \eta \prime (t)+\sigma \varsigma \prime (t)|,|g\prime (t)|]}dt-1]$$

Let *A*(ε) denote the set of points where $\left|g\prime (t)|>|f_{\text{pert}.}^{\prime }(t)\right|$. Similarly, denote the corresponding set of points where $\left|f_{\text{pert}.}^{\prime }(t)|>|g\prime (t)\right|$ by *B*(ε). We will additionally assume that the set of critical points of *f*(*t*) and *g*(*t*) are disjoint, so that χ_*A*(ε)_(*t*) ≠ 0 ⇒ *g*′(*t*) ≠ 0 and χ_*B*(ε)_(*t*) ≠ 0 ⇒ *f*′(*t*) ≠ 0. The indicator functions may be expressed in terms of Heaviside functions, *H*(*t*), as:

(30) $$\chi_{A(\varepsilon )}(t)=H(|g\prime (t)|-|f_{\text{pert}.}^{\prime }(t)|)$$

$$\chi_{B(\varepsilon )}(t)=H(|f_{\text{pert}.}^{\prime }(t)|-|g\prime (t)|)$$

Using these conventions, the first term in Equation (29) becomes:

(31) $$-\frac{1}{2}\int_{A(\varepsilon )}\frac{\left|f\prime (t)+\varepsilon \eta \prime (t)+\sigma \varsigma \prime (t)|+|g\prime (t)\right|}{\left|g\prime (t)\right|}dt-\frac{1}{2}\int_{B(\varepsilon )}\frac{\left|f\prime (t)+\varepsilon \eta \prime (t)+\sigma \varsigma \prime (t)|+|g\prime (t)\right|}{\left|f\prime (t)+\varepsilon \eta \prime (t)+\sigma \varsigma \prime (t)\right|}dt+\frac{1}{2}$$

Now,

(32) $$\frac{d|f\prime (t)+\varepsilon \eta \prime (t)+\sigma \varsigma \prime (t)|}{d\varepsilon }=\eta \prime (t)\text{sgn}(f\prime (t)+\varepsilon \eta \prime (t)+\sigma \varsigma \prime (t))$$

where we have used the operational relation for Dirac delta functions: *x*δ(*x*) = 0. In addition, if *f*′(*t*) + εη′(*t*) + σς′(*t*) ≠ 0 (which is true if *t* ∈ *B*(ε), since the set of critical points of *f*(*t*) and *g*(*t*) are disjoint), we similarly have:

(33) $$\frac{d}{d\varepsilon }\frac{1}{\left|f\prime (t)+\varepsilon \eta \prime (t)+\sigma \varsigma \prime (t)\right|}=\frac{\eta \prime (t)\text{sgn}(f\prime (t)+\varepsilon \eta \prime (t)+\sigma \varsigma \prime (t))}{{\left(f\prime (t)+\varepsilon \eta \prime (t)+\sigma \varsigma \prime (t)\right)}^{2}}$$

Using these relations, we differentiate Equation (31) with respect to ε as the first step in obtaining the variation, *V_I_*, of the first term:

(34) $$-\frac{1}{2}\int_{A(\varepsilon )}\frac{\eta \prime (t)\text{sgn}(f\prime (t)+\varepsilon \eta \prime (t)+\sigma \varsigma \prime (t))}{\left|g\prime (t)\right|}dt-\frac{1}{2}\int_{B(\varepsilon )}\frac{\eta \prime (t)\text{sgn}(f\prime (t)+\varepsilon \eta \prime (t)+\sigma \varsigma \prime (t))}{\left|f\prime (t)+\varepsilon \eta \prime (t)+\sigma \varsigma \prime (t)\right|}dt\;-\frac{1}{2}\int_{B(\varepsilon )}(|f\prime (t)+\varepsilon \eta \prime (t)+\sigma \varsigma \prime (t)|+|g\prime (t)|)[-\frac{\eta \prime (t)\text{sgn}(f\prime (t)+\varepsilon \eta \prime (t)+\sigma \varsigma \prime (t))}{{\left(f\prime (t)+\varepsilon \eta \prime (t)+\sigma \varsigma \prime (t)\right)}^{2}}]dt+\;-\frac{1}{2}{\frac{\left|f\prime (t)+\varepsilon \eta \prime (t)+\sigma \varsigma \prime (t)|+|g\prime (t)\right|}{\left|g\prime (t)\right|}|}_{\partial A(\varepsilon )}-\frac{1}{2}{\frac{\left|f\prime (t)+\varepsilon \eta \prime (t)+\sigma \varsigma \prime (t)|+|g\prime (t)\right|}{\left|f\prime (t)+\varepsilon \eta \prime (t)+\sigma \varsigma \prime (t)\right|}|}_{\partial B(\varepsilon )}$$

Now, we let ε → 0, so that Expression (34) becomes the variation for *V_I_*:

(35) $$V_{I}=-\frac{1}{2}\int_{A(0)}\frac{\eta \prime (t)\text{sgn}(f\prime (t)+\sigma \varsigma \prime (t))}{\left|g\prime (t)\right|}dt-\frac{1}{2}\int_{B(0)}\frac{\eta \prime (t)\text{sgn}(f\prime (t)+\sigma \varsigma \prime (t))}{\left|f\prime (t)+\sigma \varsigma \prime (t)\right|}dt\;-\frac{1}{2}\int_{B(0)}(|f\prime (t)+\sigma \varsigma \prime (t)|+|g\prime (t)|)[-\frac{\eta \prime (t)\text{sgn}(f\prime (t)+\sigma \varsigma \prime (t))}{{\left(f\prime (t)+\sigma \varsigma \prime (t)\right)}^{2}}]dt+\;-\frac{1}{2}{\frac{\left|f\prime (t)+\sigma \varsigma \prime (t)|+|g\prime (t)\right|}{\left|g\prime (t)\right|}|}_{\partial A(0)}-\frac{1}{2}{\frac{\left|f\prime (t)+\sigma \varsigma \prime (t)|+|g\prime (t)\right|}{\left|f\prime (t)+\sigma \varsigma \prime (t)\right|}|}_{\partial B(0)}$$

**Figure 2:**
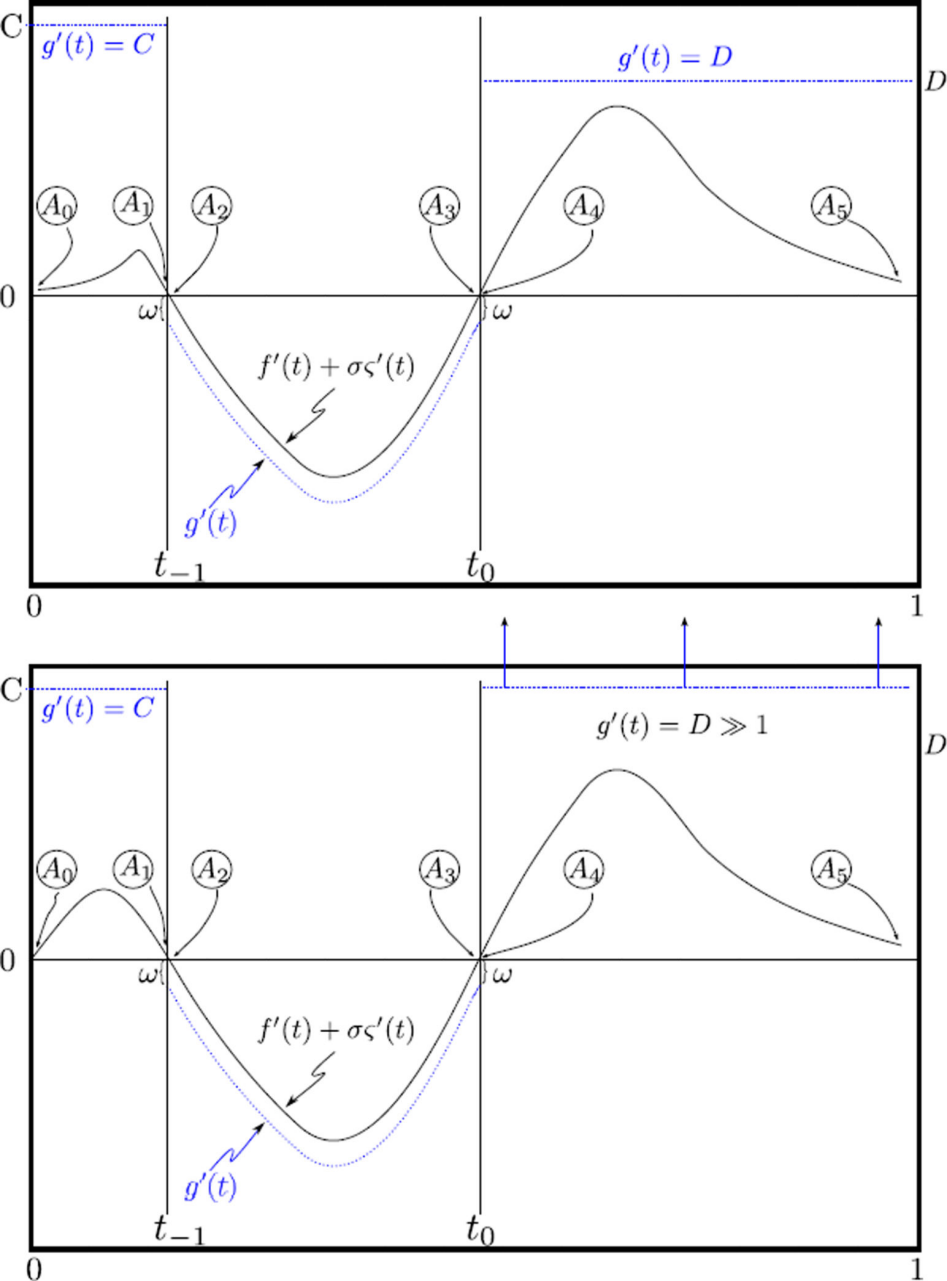
(**Top**) An example of where boundary points can be balanced exactly, *A*_0_, …, *A*_5_ ∈ ∂*A*(0), ∂*B*(0) = ∅. The cancellations may be enumerated as follows: the contribution to Equation (36) from *A*_0_, *A*_1_ cancels that from *A*_4_, *A*_5_ if $\frac{\left|f\prime (1)+\sigma \varsigma \prime (1)\right|}{\left|f\prime (0)+\sigma \varsigma \prime (0)\right|}=\frac{D}{C}$; the contribution from *A*_2_ of −1 cancels that from *A*_3_, which is +1. *C, D* can always be scaled, so that *C* > |*f*′(*t*) + σς′(*t*)|, for *t* ∈ (0, *t*_−1_), and similarly for *D* with *t* ∈ (*t*_0_, 1). The addition of additional pairs of zero-crossings for *f*′(*t*) + σς′(*t*) will lead to the same structure of canceling pairs. (**Bottom**) The case where the terms in Equation (36) from boundary points *A*_4_, *A*_5_ cannot be canceled exactly. Their contribution can, however, be made arbitrarily small by increasing the constant *D*.

Now, it is always possible to pick a reference *g*(*t*), so that at ∂*A*(0) and ∂*B*(0), we have:

(36) $$-\frac{1}{2}{\frac{\left|f\prime (t)+\sigma \varsigma \prime (t)|+|g\prime (t)\right|}{\left|g\prime (t)\right|}|}_{\partial A(0)}-\frac{1}{2}{\frac{\left|f\prime (t)+\sigma \varsigma \prime (t)|+|g\prime (t)\right|}{\left|f\prime (t)+\sigma \varsigma \prime (t)\right|}|}_{\partial B(0)}\ll 1$$

since the boundary points will either come in canceling pairs, with a contribution of ±1 at each of the boundary points (top panel of Figure 2); or there will be exact cancellations at all boundary points, but one, and the contribution from this point may be made arbitrarily small (bottom panel of Figure 2). The examples shown in Figure 2 have been chosen also to match the description of an “optimal” reference given in the Introduction after Theorem 4.

Thus, *V_I_* (Equation (35)) becomes:

(37) $$\frac{1}{2}\int_{0}^{1}\eta \prime (t)\text{sgn}(f\prime (t)+\sigma \varsigma \prime (t))[-\chi_{A}(t)\frac{1}{\left|g\prime (t)\right|}+\chi_{B}(t)\frac{\left|g\prime (t)\right|}{{\left(f\prime (t)+\sigma \varsigma \prime (t)\right)}^{2}}]dt$$

where we have written χ_*A*_(*t*) and χ_*B*_(*t*) for χ_*A*(0)_(*t*) and χ_*B*(0)_(*t*), respectively. Finally, we rewrite Equation (30):

$$\chi_{A(\varepsilon )}(t)=H(|g\prime (t)|-|f_{\text{pert}.}^{\prime }(t)|)$$

$$\chi_{B(\varepsilon )}(t)=H(|f_{\text{pert}.}^{\prime }(t)|-|g\prime (t)|)$$

in terms of *f*′(*t*), *g*′(*t*), η′(*t*) and ς′(*t*), using Equation (20), and set ε = 0 to find:

(38) $$\chi_{A}(t)=H(|g\prime (t)|-|f\prime (t)+\sigma \varsigma \prime (t)|)$$

$$\chi_{B}(t)=H(|f\prime (t)+\sigma \varsigma \prime (t)|-|g\prime (t)|)$$

This permits us to use Expression (37) to write:

(39) $$V_{I}=\frac{1}{2}\int_{0}^{1}\eta \prime (t)\text{sgn}(f\prime (t)+\sigma \varsigma \prime (t))\times \;\times [-H(|g\prime (t)|-|f\prime (t)+\sigma \varsigma \prime (t)|)\frac{1}{\left|g\prime (t)\right|}\;+H(|f\prime (t)+\sigma \varsigma \prime (t)|-|g\prime (t)|)\frac{\left|g\prime (t)\right|}{{\left(f\prime (t)+\sigma \varsigma \prime (t)\right)}^{2}}]dt$$

#### 4.1.2. Logarithmic Term

Similarly, *V_II_*, the variation for the logarithmic part of Equation (2), is obtained by first computing the derivative:

(40) $$-\frac{d}{d\varepsilon }\int_{0}^{1}\text{log }[\text{max}[|f_{\text{pert}}^{\prime }(t)|,|g\prime (t)|]]dt$$

As in the case for the first term, the variations at ∂*A*(ε) and ∂*B*(ε) cancel in pairs, since |*f*′(*t*) + εη′(*t*) + σς′(*t*)| = |*g*′(*t*)| at the boundary points; hence, the derivative is:

(41) $$-\int_{B(\varepsilon )}\frac{1}{\left|f\prime (t)+\varepsilon \eta \prime (t)+\sigma \varsigma \prime (t)\right|}\eta \prime (t)\text{sgn}(f\prime (t)+\varepsilon \eta \prime (t)+\sigma \varsigma \prime (t))dt$$

Now, we set ε = 0 to obtain the variation, *V_II_*, for the logarithmic part:

(42) $$-\int_{0}^{1}H(|f\prime (t)+\sigma \varsigma \prime (t)|-|g\prime (t)|)\frac{1}{\left|f\prime (t)+\sigma \varsigma \prime (t)\right|}\eta \prime (t)\text{sgn}(f\prime (t)+\sigma \varsigma \prime (t))dt$$

#### 4.1.3. Total Variation

**Figure 3:**
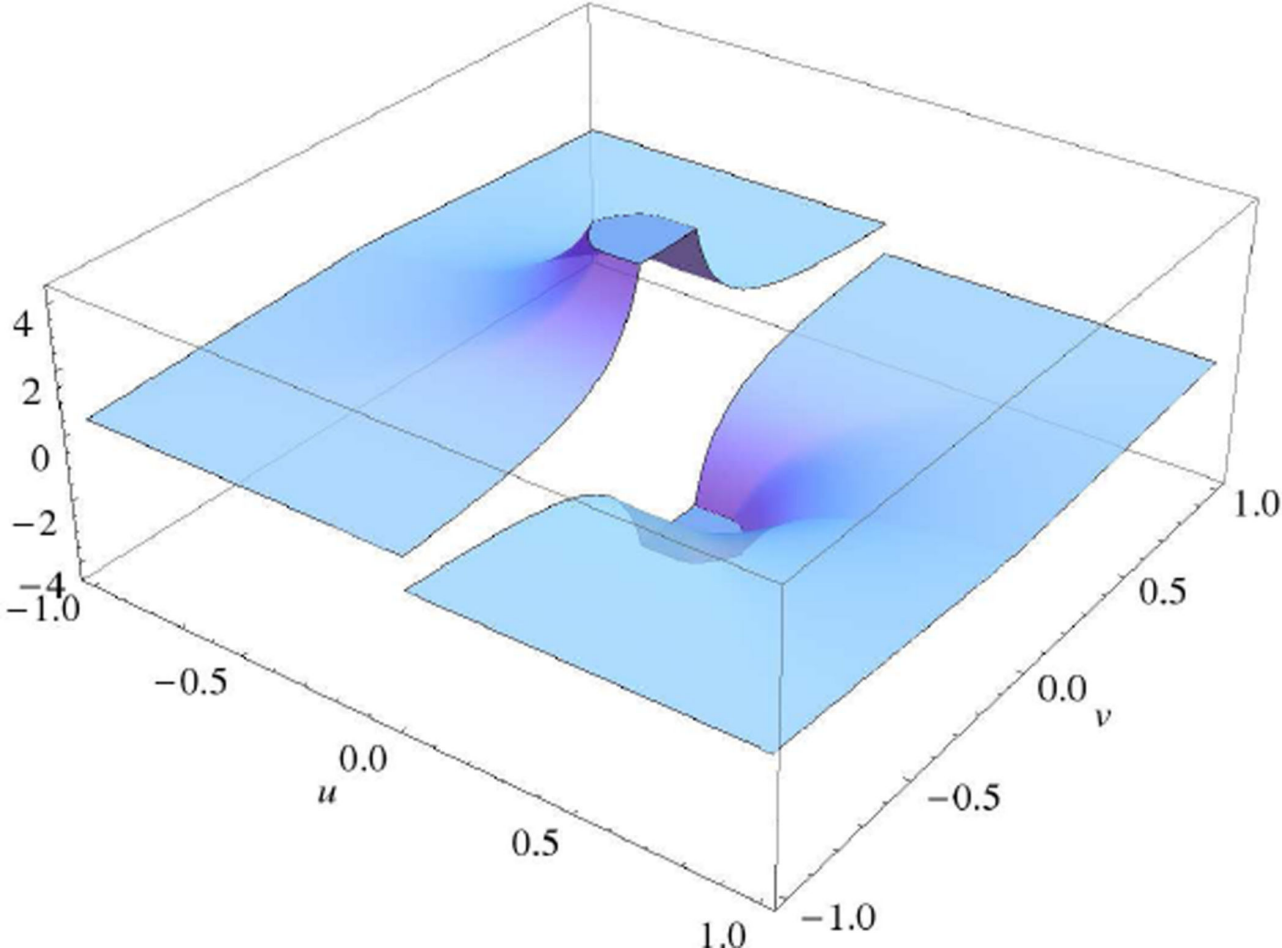
A plot of *G*(*u*, υ).

The total variation, *V*, is now:

(43) $$\delta H_{f+\sigma \varsigma ,g}[\eta ]=V_{I}+V_{II}\;=\frac{1}{2}\int_{0}^{1}\eta \prime (t)\text{sgn}(f\prime (t)+\sigma \varsigma \prime (t))[-\frac{H(|g\prime (t)|-|f\prime (t)+\sigma \varsigma \prime (t)|)}{\left|g\prime (t)\right|}\;+\frac{H(|f\prime (t)+\sigma \varsigma \prime (t)|-|g\prime (t)|)}{{\left(f\prime (t)+\sigma \varsigma \prime (t)\right)}^{2}}|g\prime (t)|-2\frac{H(|f\prime (t)+\sigma \varsigma \prime (t)|-|g\prime (t)|)}{\left|f\prime (t)+\sigma \varsigma \prime (t)\right|}]dt\;=\int_{0}^{1}\eta \prime (t)G(f\prime (t)+\sigma \varsigma \prime (t),|g\prime (t)|)dt$$

where:

(44) $$G(u,\upsilon )≔\text{sgn}(u)[-\frac{H(|\upsilon |-|u|)}{2|\upsilon |}+\frac{H(|u|-|\upsilon |)}{2u^{2}}|\upsilon |-\frac{H(|u|-|\upsilon |)}{\left|u\right|}]$$

is continuous in *u* for fixed υ and continuous in υ, except for a discontinuity across υ = 0. Figure 3 contains a plot of a typical *G*(*u*, υ). Moreover, for fixed υ, *G*(*u*, υ) is an odd function of *u*, a fact that will be significant later.

For later use, we record the alternate forms of *G*(*u*, υ):

(45) $$G(u,\upsilon )=-\frac{H(|\upsilon |-|u|)+H(|\upsilon |+|u|)-2H(u)}{2|\upsilon |}\;-H(-u-|\upsilon |)(\frac{1}{u}+\frac{\left|\upsilon \right|}{2}\frac{1}{u^{2}})-H(u-|\upsilon |)(\frac{1}{u}-\frac{\left|\upsilon \right|}{2}\frac{1}{u^{2}})$$

and:

(46) $$G(u,\upsilon )=\{\begin{array}{cc} -\frac{1}{u}-\frac{\left|\upsilon \right|}{2u^{2}}, & \text{if }u\leq -|\upsilon |\leq 0\\ \frac{1}{2|\upsilon |}, & \text{if }-|\upsilon |\leq u\leq 0\\ -\frac{1}{2|\upsilon |}, & \text{if }0\leq u\leq |\upsilon |\\ -\frac{1}{u}+\frac{\left|\upsilon \right|}{2u^{2}}, & \text{if }0\leq |\upsilon |\leq u \end{array}$$

We also record for later use the first and second partial derivatives of *G*(*u*, υ) with respect to *u*,

(47) $$\frac{\partial }{\partial u}G(u,\upsilon )=\{\begin{array}{cc} \frac{1}{u^{2}}+\frac{\left|\upsilon \right|}{u^{3}}, & \text{if }u\leq -|\upsilon |\leq 0\\ 0, & \text{if }-|\upsilon |\leq u\leq 0\\ 0, & \text{if }0\leq u\leq |\upsilon |\\ \frac{1}{u^{2}}-\frac{\left|\upsilon \right|}{u^{3}}, & \text{if }0\leq |\upsilon |\leq u \end{array}$$

which is continuous in *u* and, therefore, yields upon further differentiation with respect to *u* the distribution-free second partial derivative,

(48) $$\frac{\partial^{2}}{\partial u^{2}}G(u,\upsilon )=\{\begin{array}{cc} -\frac{2}{u^{3}}-\frac{3|\upsilon |}{u^{4}}, & \text{if }u\leq -|\upsilon |\leq 0\\ 0, & \text{if }-|\upsilon |\leq u\leq 0\\ 0, & \text{if }0\leq u\leq |\upsilon |\\ -\frac{2}{u^{3}}+\frac{3|\upsilon |}{u^{4}}, & \text{if }0\leq |\upsilon |\leq u \end{array}$$

### 4.2. Calculation of the Variation, δE_f+σς_ (η), for E_f_

We now wish to characterize the signal receiver for energy, which is given by Equation (3). The perturbed signal receiver value is:

(49) $$E_{f+\varepsilon \eta +\sigma \varsigma }(\varepsilon )=\int_{0}^{1}{\left[f(t)+\varepsilon \eta (t)+\sigma \varsigma (t)\right]}^{2}dt$$

Following the usual steps, we differentiate with respect to ε:

(50) $$\frac{dE_{f+\varepsilon \eta +\sigma \varsigma }(\varepsilon )}{d\varepsilon }=2\int_{0}^{1}[f(t)+\varepsilon \eta (t)+\sigma \varsigma (t)]\eta (t)dt.$$

The variation δ*E*_*f*+σς_ [η] is now given by:

(51) $$\delta E_{f+\sigma \varsigma }[\eta ]=\underset{\varepsilon \to 0}{\text{lim}}\frac{dE_{f+\varepsilon \eta +\sigma \varsigma }(\varepsilon )}{d\varepsilon }=2\int_{0}^{1}[f(t)+\sigma \varsigma (t)]\eta (t)dt.$$

## 5. Calculation of the Average Variations of Joint Entropy and Signal Energy

### 5.1. Calculation of the Average Variation, 〈δH_f+σς,g_(η)〉, by Wiener Integration

We now compute the average variation or expectation value of the variation over the space of noise functions, *i.e.*, we may average Equation (43) over the Brownian paths. This is obtained from the Wiener integral of $\frac{\delta H_{f,g}}{\delta f(t)}$ and is:

(52) $$\langle \delta H_{f+\sigma \varsigma ,g}[\eta ]\rangle ≔\int_{C_{0}[0,1]}\delta H_{f+\sigma \varsigma ,g}[\eta ]d_{W}x\;=\int_{C_{0}[0,1]}\int_{0}^{1}\eta \prime (t)G(f\prime (t)+\sigma \varsigma \prime (t),|g\prime (t)|])dtd_{W}x\;=\int_{C_{0}[0,1]}\int_{0}^{1}\eta \prime (t)G(f\prime (t)-\sigma \int_{0}^{1}x(t_{2})dm_{t}(t_{2}),|g\prime (t)|)dtd_{W}x$$

The function *G*(*u*, υ) is continuous in *u* for all υ; hence (Theorem 29.6 on page 443 of [40]), we can interchange the order of evaluation of integration, so:

(53) $$\int_{C_{0}[0,1]}\delta H_{f+\sigma \varsigma ,g}[\eta ]d_{W}x\;=\int_{0}^{1}\eta \prime (t)\times \int_{C_{0}[0,1]}G(f\prime (t)-\sigma \int_{0}^{1}x(t_{2})dm_{t}(t_{2}),|g\prime (t)|)d_{W}xdt$$

From Equation (128) in the Appendix, this is equal to the Lebesgue integral:

(54) $$\int_{C_{0}[0,1]}\delta H_{f+\sigma \varsigma ,g}[\eta ]d_{W}x\;=\int_{0}^{1}\frac{1}{\sqrt{2\pi }}\eta \prime (t)\int_{-\infty }^{\infty }e^{-\frac{u^{2}}{2}}G(f\prime (t)-u\sigma \|m_{t}\|,|g\prime (t)|)dudt$$

where:

(55) $$\|m_{t}\|≔\sqrt{\int_{0}^{1}m_{t}{\left(t_{2}\right)}^{2}dt_{2}}$$

Finally,

(56) $$\langle \delta H_{f+\sigma \varsigma ,g}[\eta ]\rangle =\int_{C_{0}[0,1]}\frac{\delta H_{f,g}}{\delta f(t)}d_{W}x,\;=\frac{1}{\sqrt{\pi }}\int_{0}^{1}\eta \prime (t)\int_{\infty }^{-\infty }e^{-\frac{{\left(z-f\prime (t)\right)}^{2}}{4s}}G(z,|g\prime (t)|)\frac{-1}{\sqrt{4s}}dzdt$$

where the last integral is obtained by the change of variables:

(57) $$z=f\prime (t)-(u/\sqrt{2})\sqrt{4s},\text{ where }\|m_{t}\|\sigma =\sqrt{2s}$$

This can be further simplified as:

(58) $$\langle \delta H_{f+\sigma \varsigma ,g}[\eta ]\rangle =\int_{0}^{1}\eta \prime (t)\int_{-\infty }^{\infty }[\frac{1}{\sqrt{4\pi s}}e^{-\frac{{\left(z-f\prime (t)\right)}^{2}}{4s}}]G(z,|g\prime (t)|)dzdt$$

where, based on the discussion above, typical experimental conditions will lead to σ being on the order of 10^−6^.

#### 5.1.1. The Average Variation, 〈δ*H*_*f*+σς,*g*_[η]〉, and the Heat Equation

We observe that the bracketed term in Equation (58) is the Green’s function for the heat equation [41,42], defined on ℝ, *i.e.*,

(59) $$\left[\frac{1}{\sqrt{4\pi s}}e^{-\frac{x^{2}}{4s}}\right]$$

**Figure 4:**
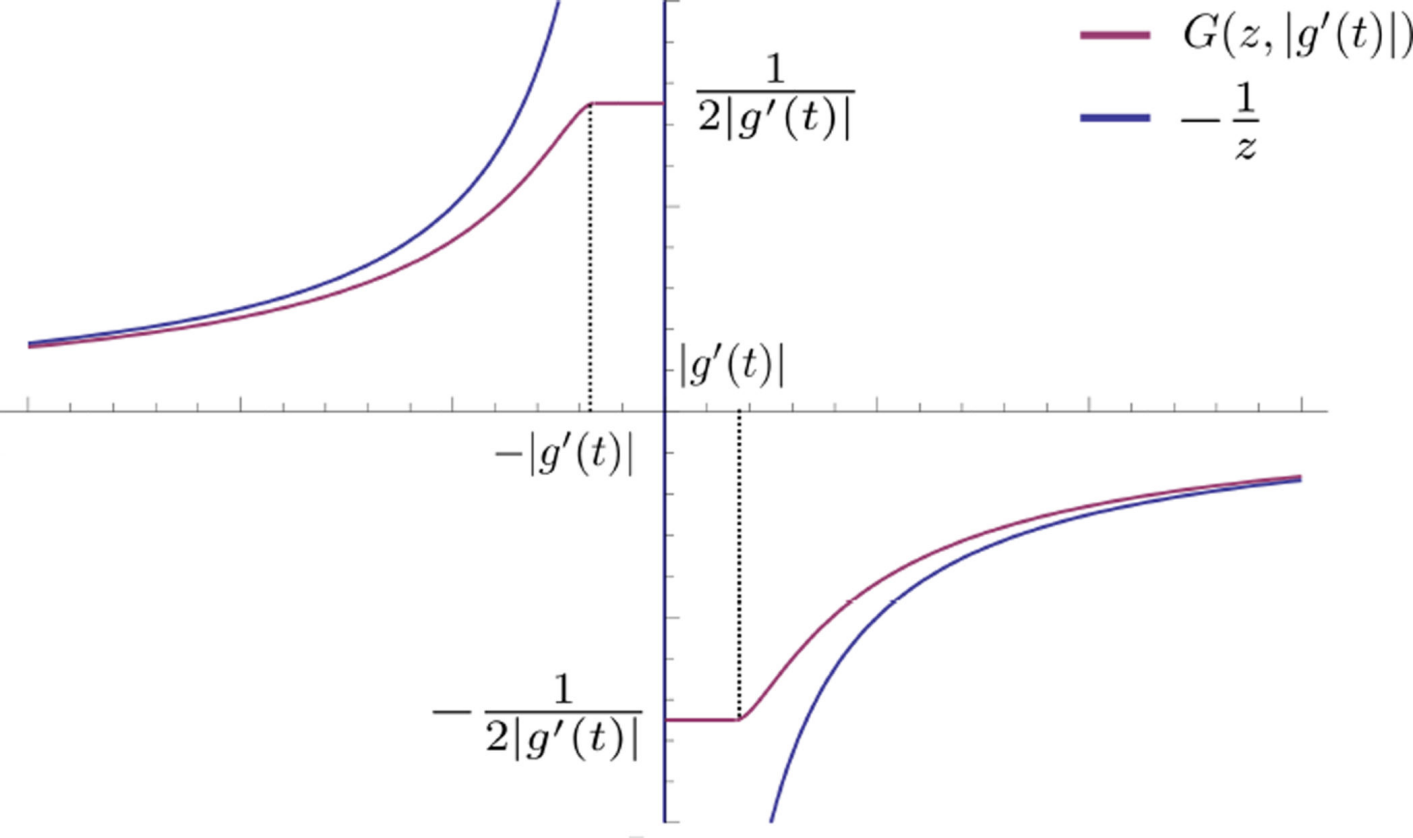
A typical initial condition from Equation (78) is shown by the purple curve. Many different initial conditions, such as the one shown, define different “heat”-surfaces, which, in turn, define the average variation 〈δ*H*_*f*+σς,*g*_[η]〉 via the inner *z*-integral in Equation (60). Also shown is the curve $y=-\frac{1}{z}$.

Thus, we have:

(60) $$\langle \delta H_{f+\sigma \varsigma ,g}[\eta ]\rangle =\int_{0}^{1}\eta \prime (t)\int_{-\infty }^{\infty }[\frac{1}{\sqrt{4\pi s}}e^{-\frac{{\left(z-f\prime (t)\right)}^{2}}{4s}}]G(z,|g\prime (t)|)dzdt,\;=\int_{0}^{1}\eta \prime (t)u_{\left|g\prime (t)\right|}(f\prime (t),s)$$

where:

(61) $$u_{\left|g\prime (t)\right|}(f\prime (t),s)≔\int_{-\infty }^{\infty }[\frac{1}{\sqrt{4\pi s}}e^{-\frac{{\left(z-f\prime (t)\right)}^{2}}{4s}}]G(z,|g\prime (t)|)dz$$

is a point on one member of a family of heat surfaces that are determined by the heat equations,

(62) $$\frac{\partial^{2}}{\partial x^{2}}u_{\left|g\prime (t)\right|}(x,s)-\frac{\partial }{\partial s}u_{\left|g\prime (t)\right|}(x,s)=0$$

with different (in fact, |*g*′(*t*)|-dependent) “initial conditions” (*i.e.*, at *s* = 0) defined by *G*(*z*, |*g*′(*t*)|). A typical example is shown in Figure 4.

Since for small noise, *s*, the heat kernel is approximated well by a Dirac delta function, we might expect that in the limit *s* → 0^+^:

(63) $$u_{\left|g\prime (t)\right|}(f\prime (t),s)\sim G(f\prime (t),|g\prime (t)|)$$

in which case, Equation (60) becomes:

(64) $$\langle \delta H_{f+\sigma \varsigma ,g}[\eta ]\rangle \sim \int_{0}^{1}\eta \prime (t)G(f\prime (t),|g\prime (t)|)dt$$

which we will shortly show is unbounded for certain choices of *g*(*t*). A good way to construct such *g*(*t*) is to pick a zero of *f*′(*t*) and have *g*′(*t*) differ only slightly from *f*′(*t*) on one side of the zero and differ greatly from it on the other side. Referring back to Equation (78), if we assume that |*g*′(*t*)| ≩ 0, then *G*(*z*, |*g*′(*t*)|) ∈ *L*^2^[ℝ], and there are a large number of theorems describing the behavior of *u*_|*g*′(*t*)|_(*x, s*). In particular, a limiting form of Theorem 5 in Chapter 5 on page 67 of [42], where the vertical sides are pushed out to ±∞, guarantees that as *s* → 0, *u*_|*g*′(*t*)|_(*z, s*) → *G*(*z*, |*g*′(*t*)|).

#### 5.1.2. The Structure of the Solution-Surfaces Associated with 〈Δ*H*_*f*+σς,*g*_[η]〉

Figure 4 also illustrates the useful inequality:

(65) $$|G(z,|g\prime (t)|)|\leq \frac{1}{2|g\prime (t)|}$$

With this result, we are now ready to prove:

##### Theorem 3

*Suppose that f is in C*^3^[0, 1] *and there exists t*_0_ ∈ (0, 1), *such that f*′(*t*_0_) = 0 *and f*″(*t*_0_) ≠ 0. *Then, there exists C*_1_ > 0, *such that for all C*_2_ > 0, *there exists a piecewise differentiable function g, independent of* η, *such that:*

(66) $$\underset{\sigma \to 0}{\text{lim}}|\langle \delta H_{f+\sigma \varsigma ,g}[\eta ]\rangle |\geq C_{2}|\eta \prime (t_{0})|-C_{1}{\left\|\eta \prime \right\|}_{\infty }$$

*In particular, provided* η′(*t*_0_) ≠ 0, *one can make* $\underset{\sigma \to 0}{\text{lim}}\langle \delta H_{f+\sigma \varsigma ,g}[\eta ]\rangle$ *arbitrarily large*.

###### Proof

We shall consider the case *f*″(*t*_0_) > 0; the other case is similar.

Choose ξ > 0 and small enough that *f*′ ∈ *C*^2^[0, 1] and strictly increasing on [*t*_0_ − ξ, *t*_0_ + ξ], so that:

(67) $$|f\prime (t_{0}+h)-hf\prime \prime (t_{0})|<\frac{f\prime \prime (t_{0})}{2}|h|,\text{ for }-\xi \leq h\leq \xi$$

We define:

(68) $$g(t)=\{\begin{array}{cc} f(t)-\omega t, & \text{if }t_{0}-\xi <t<t_{0}\\ f(t_{0})-\omega t_{0}+t-t_{0}, & \text{if }t_{0}\leq t<t_{0}+\xi \end{array}$$

where ω > 0 is chosen small.

We extend *g* to be piecewise *C*^3^[0, 1] off(*t*_0_ − ξ, *t*_0_ + ξ) and such that *g*′(*t*) ≥ 1 for all *t* ∈ [0, 1]\[*t*_0_ − ξ, *t*_0_ + ξ), except possibly finitely many points where it does not exist. The exact details of the extension do not matter, since contributions from the extension will be negligible compared to those from (*t*_0_ − ξ, *t*_0_ + ξ), as long as we do not choose a reference *g*(*t*) that does not violate the conditions discussed after Equation (36). Figure 2 illustrates how the extension might be chosen in one case.

We break the integral in Equation (64) into an integral over [*t*_0_ − ξ, *t*_0_ + ξ] and its compliment. The latter piece is bounded by:

(69) $$C_{1}{\left\|\eta \prime \right\|}_{\infty }$$

where:

(70) $$C_{1}≔\text{sup }\{G(u,\upsilon ):\upsilon \geq 1\}=1$$

by Equation (65).

The integral over [*t*_0_ − ξ, *t*_0_ + ξ] becomes:

(71) $$\eta \prime (t_{0})\int_{t_{0-\xi }}^{t_{0}}G(f\prime (t),|g\prime (t)|)dt+\eta \prime (t_{0})\int_{t_{0}}^{t_{0+\xi }}G(f\prime (t),|g\prime (t)|)dt\;=\eta \prime (t_{0})\int_{t_{0-\xi }}^{t_{0}}\frac{1}{f\prime (t)-\omega }dt-\eta \prime (t_{0})\int_{t_{0}}^{t_{0+\xi }}dt\;=\eta \prime (t_{0})\int_{t_{0-\xi }}^{t_{0}}\frac{1}{f\prime (t)-\omega }dt-\xi \eta \prime (t_{0})$$

The term ξη′(*t*_0_) may be incorporated into *C*_1_‖η′‖_∞_ by increasing *C*_1_ slightly.

By Equation (67), we have:

(72) $$|\eta \prime (t_{0})||\int_{t_{0-\xi }}^{t_{0}}\frac{1}{f\prime (t)-\omega }|\geq |\eta \prime (t_{0})|\int_{t_{0-\xi }}^{t_{0}}\frac{1}{(t_{0}-t)\frac{f\prime \prime (t_{0})}{2}+\omega }dt\;=|\eta \prime (t_{0})|\frac{2}{f\prime \prime (t_{0})}\text{log }[1+\frac{\xi f\prime \prime (t_{0})}{2\omega }]\;\sim |\eta \prime (t_{0})|\frac{2}{f\prime \prime (t_{0})}\text{log }\frac{1}{\omega }\text{as }\omega \to 0$$

If we define:

(73) $$C_{2}≔\frac{2}{f\prime \prime (t_{0})}\text{log}\frac{1}{\omega }$$

then by Equation (64):

(74) $$\underset{\sigma \to 0}{\text{lim}}\langle \delta H_{f+\sigma \varsigma ,g}[\eta ]\rangle \geq C_{2}|\eta \prime (t_{0})|-C_{1}{\left\|\eta \prime \right\|}_{\infty }$$

Finally, *g* does not depend on η. This completes the proof.

We note, however, that in order to keep the variance of the variation small, so that we maintain high sensitivity (Equation (5)), we must keep $\omega \gg \sqrt{\Delta }$ as indicated in Equation (109). This provides a practical bound on the magnitude of the variation.

We also record for later use the calculation of the maximum magnitudes of the first and second partial derivatives of *G*(*u*, υ) with respect to *u* as:

(75) $$\frac{\partial G(z,|g\prime (t)|)}{\partial z}\leq \frac{4}{27}\frac{1}{{\left|g\prime (t)\right|}^{2}}$$

and:

(76) $$\frac{\partial^{2}G(z,|g\prime (t)|)}{\partial z^{2}}\leq \frac{1}{{\left|g\prime (t)\right|}^{3}}$$

Given the structure of the integral in Equation (61), we see that *u*_|*g*′(*t*)|_(*f*′(*t*), *s*) → 0 as *s* → ∞. Moreover,

(77) $$\text{sgn}(u_{\left|g\prime (t)\right|}(f\prime (t),s))=-\text{sgn}(f\prime (t))$$

A later analysis of the signal energy *E_f_* will reveal a different “initial condition” (these “initial conditions” refer to the noise-free case), which characterizes that receiver.

The structure of *G*(*z*, |*g*′(*t*)|) is more clearly seen in Equation (46), which we recall:

(78) $$G(z,|g\prime (t)|)=\{\begin{array}{cc} -\frac{1}{z}-\frac{\left|g\prime (t)\right|}{2z^{2}}, & \text{if }z\leq -|g\prime (t)|\leq 0\\ \frac{1}{2|g\prime (t)|}, & \text{if }-|g\prime (t)|\leq z\leq 0\\ -\frac{1}{2|g\prime (t)|}, & \text{if }0\leq z\leq |g\prime (t)|\\ -\frac{1}{z}+\frac{\left|g\prime (t)\right|}{2z^{2}}, & \text{if }|g\prime (t)|\leq z \end{array}$$

We note that *G*(*z*, |*g*′(*t*)|) is determined by the mathematical form of the signal receiver, in this case *H_f,g_*, as well as the reference waveform *g*′(*t*).

**Figure 5:**
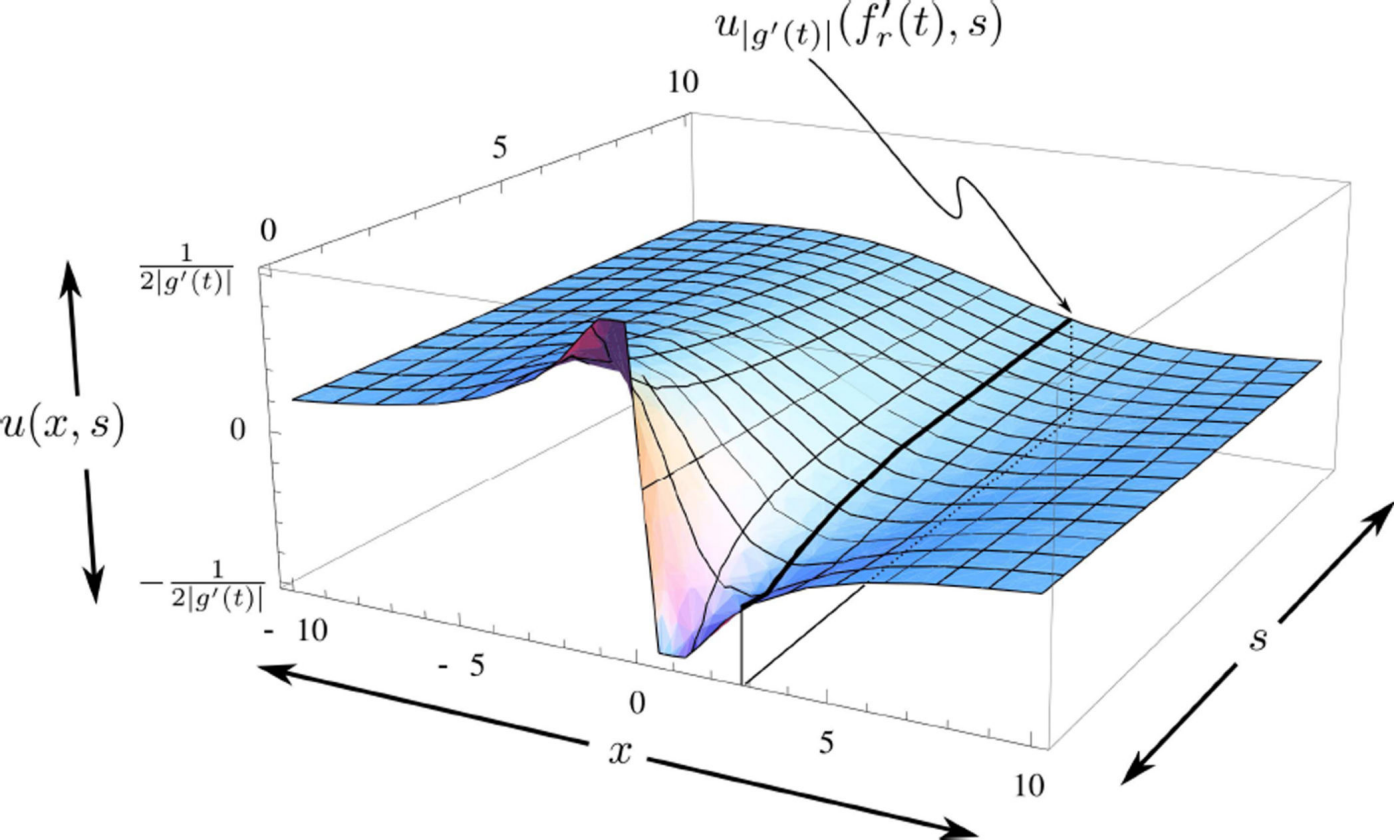
Typical solution surface for Equation (62) resulting from the initial conditions shown in Figure 4. The heavy purple line represents a typical *u*_|*g*′(*t*)|_(*f*′(*t*), *s*). Many lines, such as the one shown, each from a potentially different solution-surface (determined by different initial conditions, such as shown in Figure 4), determine the “heat”-surface that defines the average variation 〈δ*H*_*f*+σς,*g*_[η]〉 via Equation (60). The resulting surface inherits properties of the different solution surfaces, e.g., for negative values of *x*, the “heat”-surface is positive; for positive values of *x* it is negative, as *s* → ∞ the “heat”-surface decays to zero, as discussed after Equation (61). Surface shown for |*g*′(*t*)| = 0.5.

As shown in Figure 4, the supremum of the initial conditions may be made arbitrarily large by making *g*′(*t*) smaller. The effect of this change is reflected in the structure of the solution (heat) surface, as shown in Figure 5. The figure also shows that for all *z* and *s*:

(79) $$\left|u_{\left|g\prime (t)\right|}(z,s)|\leq |\frac{1}{2g\prime (t)}\right|$$

which is consistent with the maximum property for solutions to the heat equation (discussed in Chapter 2, Section 3 of [42]).

### 5.2. Calculation of the Average Variation 〈ΔE_f+σς_(η)〉

From Equation (51), the average variation is the Wiener integral:

(80) $$\langle \delta E_{f+\sigma \varsigma }[\eta ]\rangle =2\int_{C_{0}[0,1]}\int_{0}^{1}[f(t)+\sigma \varsigma (t)]\eta (t)dtd_{W}x$$

which becomes:

(81) $$\langle \delta E_{f+\sigma \varsigma }[\eta ]\rangle =2\frac{1}{\sqrt{2\pi }}\int_{-\infty }^{\infty }\int_{0}^{1}[f(t)+\sigma \|M_{t}\|u]e^{-\frac{u^{2}}{2}}\eta (t)dtdz\;=2\frac{1}{\sqrt{2\pi }}\int_{-\infty }^{\infty }\int_{0}^{1}f(t)\eta (t)e^{-\frac{u^{2}}{2}}dtdz\;=2\int_{0}^{1}f(t)\eta (t)dt$$

which has no dependence on the noise level σ and, thus, no heat surface.

We also observe that the integral in Equation (81) is the variation for the signal energy receiver in the noise-free analysis.

## 6. The Variances of the Variations

In this section, we perform the calculations required to prove:

### Theorem 4

*Suppose K*_0_ > 0 *is a constant, f*, η *are in C*^1^[0, 1] *and g is piecewise C*^1^[0, 1]*. Let* 0 < α < 1/6*. Suppose that, except at finitely many points,* |*g*′(*t*)| ≠ |*f*′(*t*)| *and* |*g*′(*t*)| ≥ *K*_0_Δ^α^.

- *Then, there exists a constant K*_1_ *that depends on* ‖η′‖_∞_*, but not on g or* σ*, such that:*
  (82) $$\text{Var}[\delta H_{f+\sigma \varsigma ,g}[\eta ]]\leq K_{1}\Delta^{1-6\alpha }\sigma^{2}+O[\Delta \sigma^{2}+\sigma^{4}]$$
- *There exists K*_2_ > 0*, that depends on* ‖η‖_∞_*, but not on* Δ *or* σ*, such that:*
  (83) $$\text{Var}[\delta E_{f+\sigma \varsigma }[\eta ]]=K_{2}\Delta^{1/2}\sigma^{2}+O[\Delta^{5/2}\sigma^{2}]$$

We break the proof into two parts.

#### Proof

Part I: Calculation of the variance of δ*H*_*f*+σς,*g*_[η]

(84) $$\text{Var}[\delta H_{f+\sigma \varsigma ,g}[\eta ]]\int_{C_{0}[0,1]}{\left[\langle \delta H_{f+\sigma \varsigma ,g}[\eta ]\rangle -\delta H_{f+\sigma \varsigma ,g}[\eta ]\right]}^{2}d_{W}x\;=\int_{C_{0}[0,1]}{\left[\delta H_{f+\sigma \varsigma ,g}[\eta ]\right]}^{2}d_{W}x-{\langle \delta H_{f+\sigma \varsigma ,g}[\eta ]\rangle }^{2}$$

Recalling Equation (43), the Wiener integral on the right-hand side of Equation (84) becomes:

(85) $$\int_{C_{0}[0,1]}{\left[\int_{0}^{1}\eta \prime (t)G(f\prime (t)+\sigma \varsigma \prime (t),|g\prime (t)|)dt\right]}^{2}d_{W}x$$

Expanding the square as a double integral (and using Equation (18) for ς′(*t*) and Equation (19) for *m_t_*), the Wiener integral may be written as:

(86) $$\int_{0}^{1}\int_{0}^{1}\eta \prime (t_{1})\eta \prime (t_{2})\times \int_{C_{0}[0,1]}G(f\prime (t_{1})-\sigma \int_{0}^{1}x(t_{3})dm_{t_{1}}(t_{3}),|g\prime (t_{1})|)\;\times G(f\prime (t_{2})-\sigma \int_{0}^{1}x(t_{3})dm_{t_{2}}(t_{3}),|g\prime (t_{2})|)d_{W}xdt_{1}dt_{2}$$

where, since *G*(*u*, υ) is jointly continuous in *u*, υ, as before, we can interchange the order of integrations. The inner Wiener integral may be replaced by a Lebesgue integral, using Equation (146). It becomes:

(87) $$\frac{1}{2\pi }\int_{-\infty }^{\infty }\int_{-\infty }^{\infty }e^{-\frac{u_{1}^{2}}{2}-\frac{u_{2}^{2}}{2}}G(f\prime (t_{1})-\sigma \|m_{t_{1}}\|u_{1},|g\prime (t_{1})|)\;\times G(f\prime (t_{2})-\sigma (u_{1}\langle m_{t_{1}},m_{t_{2}}\rangle +u_{2}n_{2}(t_{1},t_{2})),|g\prime (t_{2})|)du_{1}du_{2}$$

where (from Equation (143)):

(88) $$n_{2}(t_{1},t_{2})=\sqrt{\langle m_{t_{2}},m_{t_{2}}\rangle -\frac{{\langle m_{t_{1}},m_{t_{2}}\rangle }^{2}}{\langle m_{t_{1}},m_{t_{1}}\rangle }}$$

We also point out for later use that calculation of the maximum of *n*_2_(*t*_1_, *t*_2_)^2^ shows:

(89) $$n_{2}{\left(t_{1},t_{2}\right)}^{2}\leq 1$$

Changing variables using:

(90) $$z_{1}=f\prime (t_{1})-\sqrt{2s_{1}}u_{1},\text{ where }\sigma \|m_{t_{1}}\|=\sqrt{2s_{1}}$$

$$z_{2}=f\prime (t_{2})-\sqrt{2s_{2}}u_{2},\text{ where }\sigma n_{2}(t_{1},t_{2})=\sqrt{2s_{2}}$$

permits the integral in Expression (87) to be rewritten as:

(91) $$\int_{-\infty }^{\infty }\int_{-\infty }^{\infty }\frac{1}{\sqrt{4\pi s_{1}}}\frac{1}{\sqrt{4\pi s_{2}}}e^{-\frac{{\left(z_{1}-f\prime (t_{1})\right)}^{2}}{4s_{1}}-\frac{{\left(z_{2}-f\prime (t_{2})\right)}^{2}}{4s_{2}}}\;\times G(z_{1},|g\prime (t_{1})|)G(z_{2}+(z_{1}-f\prime (t_{1}))\frac{\langle m_{t_{1}},m_{t_{2}}\rangle }{\left\|m_{t_{1}}\right\|},|g\prime (t_{2})|)dz_{1}dz_{2}$$

Substituting this into Equation (84), we obtain:

(92) $$\text{Var}[\delta H_{f+\sigma \varsigma ,g}[\eta ]]\;=\int_{0}^{1}\int_{0}^{1}\eta \prime (t_{1})\eta \prime (t_{2})\;\times \int_{-\infty }^{\infty }\int_{-\infty }^{\infty }\frac{1}{\sqrt{4\pi s_{1}}}\frac{1}{\sqrt{4\pi s_{2}}}e^{-\frac{{\left(z_{1}-f\prime (t_{1})\right)}^{2}}{4s_{1}}-\frac{{\left(z_{2}-f\prime (t_{2})\right)}^{2}}{4s_{2}}}\;\times G(z_{1},|g\prime (t_{1})|)G(z_{2}+(z_{1}-f\prime (t_{1}))\frac{\langle m_{t_{1}},m_{t_{2}}\rangle }{\left\|m_{t_{1}}\right\|},|g\prime (t_{2})|)\;\times dz_{1}dz_{2}dt_{1}dt_{2}\;-{\left[\int_{0}^{1}\eta \prime (t)\int_{-\infty }^{\infty }[\frac{1}{\sqrt{4\pi s}}e^{-\frac{{\left(z-f\prime (t)\right)}^{2}}{4s}}]G(z,|g\prime (t)|)dzdt\right]}^{2}$$

As a double check of the result, we observe that as *s*_1_, *s*_2_ → 0^+^, the heat-kernels in the first and second terms approach Dirac delta functions centered at *f*′(*t*), *f*′(*t*_1_) and *f*′(*t*_2_), *i.e.*, δ(*z* − *f*′(*t*)), δ(*z*_1_ − *f*′(*t*_1_)) and δ(*z*_2_ − *f*′(*t*_2_)), respectively, and:

(93) $$\underset{s_{1},s_{2}\to 0^{+}}{\text{lim}}\text{Var}[\delta H_{f+\sigma \varsigma ,g}[\eta ]]=0$$

as expected.

We now rewrite the right-hand side of Equation (92) in terms of solutions of the heat equation, as was done following Equation (60), and find that Equation (92) equals:

(94) $$\int_{0}^{1}dt_{1}\eta \prime (t_{1})\int_{0}^{1}dt_{2}\eta \prime (t_{2})\Lambda (t_{1},t_{2},s_{1},s_{2})-{\left[\int_{0}^{1}dt_{1}\eta \prime (t)u_{\left|g\prime (t)\right|}(f\prime (t),s)\right]}^{2}$$

where:

(95) $$\Lambda (t_{1},t_{2},s_{1},s_{2})≔\int_{-\infty }^{\infty }dz_{1}\frac{1}{\sqrt{4\pi s_{1}}}e^{-\frac{{\left(z_{1}-f\prime (t_{1})\right)}^{2}}{4s_{1}}}G(z_{1},|g\prime (t_{1})|)\;\times \int_{-\infty }^{\infty }dz_{2}\frac{1}{\sqrt{4\pi s_{2}}}e^{-\frac{{\left(z_{2}-f\prime (t_{2})\right)}^{2}}{4s_{2}}}\;\times G(z_{2}+(z_{1}-f\prime (t_{1}))\frac{\langle m_{t_{1}},m_{t_{2}}\rangle }{\left\|m_{t_{1}}\right\|},|g\prime (t_{2})|)\;=\int_{-\infty }^{\infty }dz_{1}\frac{1}{\sqrt{4\pi s_{1}}}e^{-\frac{{\left(z_{1}-f\prime (t_{1})\right)}^{2}}{4s_{1}}}G(z_{1},|g\prime (t_{1})|)\;\times u_{\left|g\prime (t_{2})\right|}(f\prime (t_{2})+(z_{1}-f\prime (t_{1}))\frac{\langle m_{t_{1}},m_{t_{2}}\rangle }{\left\|m_{t_{1}}\right\|},s_{2})$$

where we have used the change of variables:

(96) $$\xi =z_{2}+(z_{1}-f\prime (t_{1}))\frac{\langle m_{t_{1}},m_{t_{2}}\rangle }{\left\|m_{t_{1}}\right\|}$$

and Equation (61) to go from the first to the second equations.

We now observe that, by Equation (22), 〈*m*_*t*_1__, *m*_*t*_2__〉 = 0 when |*t*_1_ − *t*_2_| > Δ, in which case:

(97) $$\Lambda (t_{1},t_{2},s_{1},s_{2})=\int_{-\infty }^{\infty }dz_{1}\frac{1}{\sqrt{4\pi s_{1}}}e^{-\frac{{\left(z_{1}-f\prime (t_{1})\right)}^{2}}{4s_{1}}}G(z_{1},|g\prime (t_{1})|)\;\times \int_{-\infty }^{\infty }dz_{2}\frac{1}{\sqrt{4\pi s_{2}}}e^{-\frac{{\left(z_{2}-f\prime (t_{2})\right)}^{2}}{4s_{2}}}G(z_{2},|g\prime (t_{2})|)\;=u_{\left|g\prime (t_{1})\right|}(f\prime (t_{1}),s_{1})u_{\left|g\prime (t_{2})\right|}(f\prime (t_{2}),s_{2})$$

and so, Equation (94) may be written as:

(98) $$\text{Var }[\delta H_{f+\sigma \varsigma ,g}[\eta ]]\;=\int_{0}^{1}dt_{1}\eta \prime (t_{1})\int_{0}^{1}dt_{2}\eta \prime (t_{2})u_{\left|g\prime (t_{1})\right|}(f\prime (t_{1}),s_{1})u_{\left|g\prime (t_{2})\right|}(f\prime (t_{2}),s_{2})\;-\int_{0}^{1}dt_{1}\eta \prime (t_{1})\int_{t_{1}-\Delta }^{t_{1}+\Delta }dt_{2}\eta \prime (t_{2})[u_{\left|g\prime (t_{1})\right|}(f\prime (t_{1}),s_{1})u_{\left|g\prime (t_{2})\right|}(f\prime (t_{2}),s_{2})]\;+\int_{0}^{1}dt_{1}\eta \prime (t_{1})\int_{t_{1}-\Delta }^{t_{1}+\Delta }dt_{2}\eta \prime (t_{2})\Lambda (t_{1},t_{2},s_{1},s_{2}).\;-{\left[\int_{0}^{1}dt_{1}\eta \prime (t)u_{\left|g\prime (t)\right|}(f\prime (t),s)\right]}^{2}$$

The functions appearing in Equation (98) are based on integrals that have the form:

(99) $$\frac{1}{\sqrt{4\pi s_{i}}}\int_{-\infty }^{\infty }e^{-\frac{{\left(z-a\right)}^{2}}{4s_{i}}}h(z)dz\sim h(a)+s_{i}h\prime \prime (a)+O[s_{i}^{2}]h^{\left(4\right)}(a)$$

where limiting behavior as *s_i_* → 0 for *i* = 1, 2 may be found using a Laplace expansion, as described in Equation 6.4.35 of Bender and Orszag [43], and Equation (99) holds, provided *h* is *C*^5^ in a neighborhood of *a*.

Consequently, we see that, after rewriting the first integral as a product of a *t*_1_ and a *t*_2_ integral and using Equations (90), the difference of the first and the last terms in Equation (98) is of order σ^4^ in the limit *s*_1_, *s*_2_ → 0 and may therefore be dropped.

Consequently, we focus attention on the remaining integrals. Equation (99) enables this refinement of the limiting form of Equation (63):

(100) $$u_{\left|g\prime (t)\right|}(f\prime (t),s)=\int_{-\infty }^{\infty }\frac{1}{\sqrt{4\pi s}}e^{-\frac{{\left(z-f\prime (t)\right)}^{2}}{4s}}G(z,|g\prime (t)|)dz\;\sim G(f\prime (t),|g\prime (t)|)+sG^{\left(2\right)}(f\prime (t),|g\prime (t)|)\;+O[s_{i}^{2}]G^{\left(4\right)}(f\prime (t),|g\prime (t)|)$$

where the *n*-th-partial derivative of *G*(*u*, υ) with respect to the first argument is denoted by *G*^(*n*)^(*u*, υ) and is guaranteed to exist for all *n* at all but finitely many points by the assumption that |*g*′(*t*)| ≠ |*f*′(*t*)| at all but finitely many points. Moreover, since |*g*′(*t*)| is bounded away from zero by hypothesis, there is a uniform bound on *G*^(4)^ (*f*′(*t*), |*g*′(*t*)|) off the set of points where |*g*′(*t*)| = |*f*′(*t*)|.

Consider the second integral in Equation (98). The term in brackets becomes (ignoring those finitely many points where *G*(*f*′(*t*), |*g*′(*t*)|) might be non-smooth, because |*f*′(*t*)| = |*g*′(*t*)|), and using Equation (90) to replace *s*_1_, *s*_2_:

(101) $$\left[u_{\left|g\prime (t_{1})\right|}(f\prime (t_{1}),s_{1})u_{\left|g\prime (t_{2})\right|}(f\prime (t_{2}),s_{2})]\sim G(f\prime (t_{1}),|g\prime (t_{1})|)G(f\prime (t_{2}),|g\prime (t_{2})|)+\frac{\sigma^{2}}{2}G(f\prime (t_{1}),|g\prime (t_{1})|)n_{2}{\left(t_{1},t_{2}\right)}^{2}G^{\left(2\right)}(f\prime (t_{2}),|g\prime (t_{2})|)+\frac{\sigma^{2}}{2}{\left\|m_{t_{1}}\right\|}^{2}G^{\left(2\right)}(f\prime (t_{1}),|g\prime (t_{1})|)G(f\prime (t_{2}),|g\prime (t_{2})|)+O[\sigma^{4}\right]$$

We next consider the third integral in Equation (98), by Equations (95) and (100) as *s*_2_ → 0,

(102) $$\Lambda (t_{1},t_{2},s_{1},s_{2})\sim \int_{-\infty }^{\infty }dz_{1}\frac{1}{\sqrt{4\pi s_{1}}}e^{-\frac{{\left(z_{1}-f\prime (t_{1})\right)}^{2}}{4s_{1}}}G(z_{1},|g\prime (t_{1})|)[\Phi_{1}(z_{1})+s_{2}\Phi_{2}(z_{1})]$$

with:

(103) $$\Phi_{1}(z_{1})+s_{2}\Phi_{2}(z_{1})≔G(f\prime (t_{2})+(z_{1}-f\prime (t_{1}))\frac{\langle m_{t_{1}},m_{t_{2}}\rangle }{\left\|m_{t_{1}}\right\|},|g\prime (t_{2})|)\;+s_{2}G^{\left(2\right)}(f\prime (t_{2})+(z_{1}-f\prime (t_{1}))\frac{\langle m_{t_{1}},m_{t_{2}}\rangle }{\left\|m_{t_{1}}\right\|},|g\prime (t_{2})|)$$

defined as indicated to simplify notation. Applying Equation (99) to Equation (102), we find that Λ(*t*_1_, *t*_2_, *s*_1_, *s*_2_) is asymptotic to:

(104) $$G(f\prime (t_{1}),|g\prime (t_{1})|)[\Phi_{1}(f\prime (t_{1}))+s_{2}\Phi_{2}(f\prime (t_{1}))]\;+s_{1}G^{\left(2\right)}(f\prime (t_{1}),|g\prime (t_{1})|)[\Phi_{1}(f\prime (t_{1}))+s_{2}\Phi_{2}(f\prime (t_{1}))]\;+2s_{1}G^{\left(1\right)}(f\prime (t_{1}),|g\prime (t_{1})|)[\Phi_{1}^{\left(1\right)}(f\prime (t_{1}))+s_{2}\Phi_{2}^{\left(1\right)}(f\prime (t_{1}))]\;+s_{1}G(f\prime (t_{1}),|g\prime (t_{1})|)[\Phi_{1}^{\left(2\right)}(f\prime (t_{1}))+s_{2}\Phi_{2}^{\left(2\right)}(f\prime (t_{1}))]+O[\sigma^{4}]$$

Since $\Phi_{1}^{\left(n\right)}(f\prime (t_{1}))=G^{\left(n\right)}(f\prime (t_{2}),|g\prime (t_{2})|)$ and $\Phi_{2}^{\left(n\right)}(f\prime (t_{1}))=G^{\left(n+2\right)}(f\prime (t_{2}),|g\prime (t_{2})|)$, Λ(*t*_1_, *t*_2_, *s*_1_, *s*_2_) becomes:

(105) $$\Lambda (t_{1},t_{2},s_{1},s_{2})\sim G(f\prime (t_{1}),|g\prime (t_{1})|)G(f\prime (t_{2}),|g\prime (t_{2})|)\;+s_{2}G(f\prime (t_{1}),|g\prime (t_{1})|)G^{\left(2\right)}(f\prime (t_{2}),|g\prime (t_{2})|)\;+s_{1}G^{\left(2\right)}(f\prime (t_{1}),|g\prime (t_{1})|)G(f\prime (t_{2}),|g\prime (t_{2})|)\;+2s_{1}G^{\left(1\right)}(f\prime (t_{1}),|g\prime (t_{1})|)G^{\left(1\right)}(f\prime (t_{2}),|g\prime (t_{2})|)\;+s_{1}G(f\prime (t_{1}),|g\prime (t_{1})|)G^{\left(2\right)}(f\prime (t_{2}),|g\prime (t_{2})|)+O[\sigma^{4}]$$

where we used Equation (90) to write the error term as a function of σ.

Using (105) and (90), the difference between the second and third integrals appearing in Equation (98) gives:

(106) $$\text{Var }[\delta H_{f+\sigma \varsigma ,g}[\eta ]]=\frac{\sigma^{2}}{2}\int_{0}^{1}dt_{1}\eta \prime (t_{1})\int_{t_{1}-\Delta }^{t_{1}+\Delta }dt_{2}\eta \prime (t_{2})\;\{G(f\prime (t_{1}),|g\prime (t_{1})|)n{\left(m_{t_{1}},m_{t_{2}}\right)}^{2}G^{\left(2\right)}(f\prime (t_{2}),|g\prime (t_{2})|)\;+2{\left\|m_{t_{1}}\right\|}^{2}G^{\left(1\right)}(f\prime (t_{1}),|g\prime (t_{1})|)G^{\left(1\right)}(f\prime (t_{2}),|g\prime (t_{2})|)\;+{\left\|m_{t_{1}}\right\|}^{2}\eta \prime (t_{1})G(f\prime (t_{1}),|g\prime (t_{1})|)G^{\left(2\right)}(f\prime (t_{2}),|g\prime (t_{2})|)\;-G(f\prime (t_{1}),|g\prime (t_{1})|){\left\|m_{t_{2}}\right\|}^{2}G^{\left(2\right)}(f\prime (t_{2}),|g\prime (t_{2})|)\}$$

to accuracy *O*[σ^4^]. This may be further simplified, introducing errors of *O*[2Δ] by truncating the upper bound of integration for *t*_1_ in the last two integrals from 1 to 1 − 2Δ, so that by Equation (23), ‖*m_t_*‖ = 1, and the last two pieces cancel, leaving:

$$\text{Var }[\delta H_{f+\sigma \varsigma ,g}[\eta ]]=\;+\frac{\sigma^{2}}{2}\int_{0}^{1}dt_{1}\eta \prime (t_{1})G(f\prime (t_{1}),|g\prime (t_{1})|)\int_{t_{1}-\Delta }^{t_{1}+\Delta }dt_{2}\eta \prime (t_{2})n{\left(m_{t_{1}},m_{t_{2}}\right)}^{2}G^{\left(2\right)}(f\prime (t_{2}),|g\prime (t_{2})|)\;+\sigma^{2}\int_{0}^{1}dt_{1}{\left\|m_{t_{1}}\right\|}^{2}\eta \prime (t_{1})G^{\left(1\right)}(f\prime (t_{1}),|g\prime (t_{1})|)\int_{t_{1}-\Delta }^{t_{1}+\Delta }dt_{2}\eta \prime (t_{2})G^{\left(1\right)}(f\prime (t_{2}),|g\prime (t_{2})|)+O[\sigma^{4}]$$

Focusing on the product of integrals in the second row, we see that at the cost of an additional error term of *O*[Δ], we may replace ‖*m_t_*‖ by 1 in the *t*_1_ integral and then use Schwartz’s inequality to bound the integral by the norms shown. To bound the *t*_2_ integral, we use Equations (17) and (75) to obtain:

(107) $$\text{Var }[\delta H_{f+\sigma \varsigma ,g}[\eta ]]\;\leq +\frac{\sigma^{2}}{2}\int_{0}^{1}dt_{1}\eta \prime (t_{1})G(f\prime (t_{1}),|g\prime (t_{1})|)\int_{t_{1}-\Delta }^{t_{1}+\Delta }dt_{2}\eta \prime (t_{2})n{\left(m_{t_{1}},m_{t_{2}}\right)}^{2}G^{\left(2\right)}(f\prime (t_{2}),|g\prime (t_{2})|)\;+\sigma^{2}{\left\|\eta \prime \right\|}^{2}{\left\|G^{\left(1\right)}\right\|}^{2}\int_{t_{1}-\Delta }^{t_{1}+\Delta }dt_{2}{\left\|\eta \prime \right\|}_{\infty }\frac{4}{27}\frac{1}{{\left|g\prime (t_{2})\right|}^{2}}+O[\sigma^{4}]$$

Focusing next on the integrals in the first row of the inequality, we use Schwartz’s inequality to bound the *t*_1_ integral and Equations (17), (76) and (89) to bound the *t*_2_ integral to obtain:

(108) $$\text{Var }[\delta H_{f+\sigma \varsigma ,g}[\eta ]]\;\leq \frac{\sigma^{2}}{2}{\left\|\eta \prime \right\|}^{2}{\left\|G\right\|}^{2}{\left\|\eta \prime \right\|}_{\infty }\int_{t_{1}-\Delta }^{t_{1}+\Delta }dt_{2}\frac{1}{{\left|g\prime (t_{2})\right|}^{3}}\;+\sigma^{2}{\left\|\eta \prime \right\|}^{2}{\left\|G^{\left(1\right)}\right\|}^{2}{\left\|\eta \prime \right\|}_{\infty }\frac{4}{27}\int_{t_{1}-\Delta }^{t_{1}+\Delta }dt_{2}\frac{1}{{\left|g\prime (t_{2})\right|}^{2}}+O[\sigma^{2}]O[\Delta ]+O[\sigma^{4}].$$

Since the reference satisfies the constraint:

(109) $$|g\prime (t)|\geq K_{0}\Delta^{\alpha }$$

where α > 0, then we have:

(110) $$\text{Var}[\delta H_{f+\sigma \varsigma ,g}[\eta ]]\leq \frac{\sigma^{2}}{2}{\left\|\eta \prime \right\|}_{\infty }^{3}{\left(K_{0}\Delta^{-\alpha }\right)}^{2}\Delta K_{0}^{3}\Delta^{-3\alpha }\;+\sigma^{2}{\left\|\eta \prime \right\|}_{\infty }^{3}{\left(\frac{4}{27}K_{0}^{2}\Delta^{-2\alpha }\right)}^{2}\frac{4}{27}\Delta K_{0}^{2}\Delta^{-2\alpha }\;+O[\sigma^{2}]O[\Delta ]+O[\sigma^{4}]$$

This simplifies to our final bound:

(111) $$0<\text{Var }[\delta H_{f+\sigma \varsigma ,g}[\eta ]]\leq K_{3}{\left\|\eta \prime \right\|}_{\infty }^{3}\sigma^{2}\Delta^{1-6\alpha }+O[\sigma^{4}]$$

where $K_{3}=K_{0}^{6}{\left(4/27\right)}^{3}$ is a constant independent of *g*, η, Δ and σ. Let $K_{1}≔K_{3}{\left\|\eta \prime \right\|}_{\infty }^{3}$, and we are done.

Part II: Calculation of the variance of δ*E*_*f*+σς_ [η] The variance of the variation, δ*E*_*f*+σς_ [η], is:

(112) $$\text{Var }[\delta E_{f+\sigma \varsigma }[\eta ]]=\int_{C_{0}[0,1]}{\left[\delta E_{f+\sigma \varsigma }[\eta ]\right]}^{2}d_{W}x-{\langle \delta E_{f+\sigma \varsigma }[\eta ]\rangle }^{2}$$

Recalling Equations (81) and (51) and using Equation (13) for ς(*t*) and Equation (16) for *M_t_*, the Wiener integral on the right-hand side of Equation (112) becomes:

(113) $$\int_{C_{0}[0,1]}{\left[2\int_{0}^{1}[f(t)+\sigma \varsigma (t)]\eta (t)dt\right]}^{2}d_{W}x\;=2\int_{0}^{1}du_{1}2\int_{0}^{1}du_{1}\eta (t_{1})\eta (t_{2})\;\times \int_{C_{0}[0,1]}d_{W}x[(f(t_{1})+\sigma \int_{0}^{1}x_{2}(t_{3})dM_{t_{1}}(t_{3}))\;\times (f(t_{2})+\sigma \int_{0}^{1}x_{2}(t_{3})dM_{t_{2}}(t_{3}))]$$

Using Equation (146), the right-hand side may be written in terms of Lebesgue integrals as:

(114) $$2\int_{0}^{1}dt_{1}2\int_{0}^{1}dt_{1}\eta (t_{1})\eta (t_{2})\frac{1}{2\pi }\int_{-\infty }^{\infty }e^{-\frac{u_{1}^{2}}{2}}du_{1}\int_{-\infty }^{\infty }e^{-\frac{u_{2}^{2}}{2}}du_{2}\;\times [(f(t_{1})+\sigma \|M_{t_{1}}\|u_{1})(f(t_{2})+\sigma (\langle M_{t_{1}},M_{t_{2}}\rangle u_{1}+N_{2}(t_{1},t_{2})u_{2}))]$$

where (from Equation (143)):

(115) $$N_{2}(t_{1},t_{2})=\sqrt{\langle M_{t_{2}},M_{t_{2}}\rangle -\frac{{\langle M_{t_{1}},M_{t_{2}}\rangle }^{2}}{\langle M_{t_{1}},M_{t_{1}}\rangle }}$$

Changing variables using:

(116) $$f(t_{1})-z_{1}=-\sqrt{2s_{1}}u_{1},\text{ where }\sigma \|M_{t_{1}}\|=\sqrt{2s_{1}}$$

$$f(t_{2})-z_{2}=-\sqrt{2s_{2}}u_{2},\text{ where }\sigma N_{2}(t_{1},t_{2})=\sqrt{2s_{2}}$$

permits Expression (114) to be rewritten as:

$$2\int_{0}^{1}2\int_{0}^{1}\eta (t_{1})\eta (t_{2})\frac{1}{\sqrt{4\pi s_{1}}}\int_{-\infty }^{\infty }e^{-\frac{{\left(z_{1}-f(t_{1})\right)}^{2}}{4s_{1}}}\frac{1}{\sqrt{4\pi s_{2}}}\int_{-\infty }^{\infty }e^{-\frac{{\left(z_{2}-f(t_{2})\right)}^{2}}{4s_{2}}}\;\times [z_{1}(z_{2}+(z_{1}-f(t_{1}))\frac{\langle M_{t_{1}},M_{t_{2}}\rangle }{\left\|M_{t_{1}}\right\|})]dz_{2}dz_{1}dt_{2}dt_{1}$$

Using Equation (28), this becomes:

(117) $$4\int_{0}^{1}\int_{0}^{1}\eta (t_{1})\eta (t_{2})\frac{1}{\sqrt{4\pi s_{1}}}\int_{-\infty }^{\infty }e^{-\frac{{\left(z_{1}-f(t_{1})\right)}^{2}}{4s_{1}}}\;\times \frac{1}{\sqrt{4\pi s_{2}}}\int_{-\infty }^{\infty }e^{-\frac{{\left(z_{2}-f(t_{2})\right)}^{2}}{4s_{2}}}z_{1}z_{2}dz_{2}dz_{1}dt_{2}dt_{1}+\sqrt{\Delta }\Omega$$

where:

(118) $$\Omega =4\int_{0}^{1}\int_{0}^{1}\eta (t_{1})\eta (t_{2})\frac{1}{\sqrt{4\pi s_{1}}}\int_{-\infty }^{\infty }e^{-\frac{{\left(z_{1}-f(t_{1})\right)}^{2}}{4s_{1}}}\frac{1}{\sqrt{4\pi s_{2}}}\int_{-\infty }^{\infty }e^{-\frac{{\left(z_{2}-f(t_{2})\right)}^{2}}{4s_{2}}}\;\times z_{1}(z_{1}-f(t_{1}))\frac{\text{min }(t_{1},t_{2})}{\sqrt{t_{1}}}dz_{2}dz_{1}dt_{2}dt_{1}+O[\Delta^{2}]\sigma^{2}$$

Note that the first term in Equation (118) may be calculated exactly as:

(119) $$4\int_{0}^{1}\int_{0}^{1}\eta (t_{1})\eta (t_{2})f(t_{1})f(t_{2})dt_{1}dt_{2}$$

which, by Equation (81), cancels the −〈δ*E*_*f*+σς_ [η]〉^2^ appearing in Equation (112).

We now rewrite Ω as:

(120) $$\Omega =4\int_{0}^{1}\int_{0}^{1}\eta (t_{1})\eta (t_{2})\frac{1}{\sqrt{4\pi s_{1}}}\int_{-\infty }^{\infty }e^{-\frac{{\left(z_{1}-f(t_{1})\right)}^{2}}{4s_{1}}}\frac{1}{\sqrt{4\pi s_{2}}}\int_{-\infty }^{\infty }e^{-\frac{{\left(z_{2}-f(t_{2})\right)}^{2}}{4s_{2}}}\;\times {\left(z_{1}-f(t_{1})\right)}^{2}\frac{\text{min }(t_{1},t_{2})}{\sqrt{t_{1}}}dz_{2}dz_{1}dt_{2}dt_{1}\;+4\int_{0}^{1}\int_{0}^{1}\eta (t_{1})\eta (t_{2})\frac{1}{4\pi s_{1}}\int_{-\infty }^{\infty }e^{-\frac{{\left(z_{1}-f(t_{1})\right)}^{2}}{4s_{1}}}\frac{1}{\sqrt{4\pi s_{2}}}\int_{-\infty }^{\infty }e^{-\frac{{\left(z_{2}-f(t_{2})\right)}^{2}}{4s_{2}}}\;\times f(t_{1})(z_{1}-f(t_{1}))\frac{\text{min }(t_{1},t_{2})}{\sqrt{t_{1}}}dz_{2}dz_{1}dt_{2}dt_{1}+O[\Delta^{2}]\sigma^{2}$$

In the second integral the *z*_1_ integration is of an odd integrand over a symmetric interval and, hence, vanishes, so that we obtain after some rearrangement and simplification of the remaining Gaussian integrals:

(121) $$\Omega =4\int_{0}^{1}\int_{0}^{1}\frac{\text{min }(t_{1},t_{2})}{\sqrt{t_{1}}}\eta (t_{1})\eta (t_{2})s_{2}dt_{2}dt_{1}+O[\Delta^{2}]\sigma^{2}$$

(122) $$=2\sqrt{2}\sigma^{2}\int_{0}^{1}\int_{0}^{1}\frac{\text{min }(t_{1},t_{2})}{\sqrt{t_{1}}}\eta (t_{1})\eta (t_{2})N_{2}(t_{1},t_{2})dt_{2}dt_{1}+O[\Delta^{2}]\sigma^{2}$$

where we have used Equation (116) to obtain the last equation.

Inserting the limiting form of the integral into Expression (117) of small positive *s*_1_, *s*_2_ into Equation (112) and using Equation (119), we obtain:

(123) $$\text{Var}[\delta E_{f+\sigma \varsigma }[\eta ]]=K_{2}\Delta^{1/2}\sigma^{2}+O(\Delta^{5/2}\sigma^{2})$$

where *K*_2_ is a constant independent of Δ and σ, but depending on η.

This completes the proof of the theorem.

## 7. A Short List of Wiener Integrals

### 7.1. The First Wiener Integral

The first type of Wiener integral is described by the following:

#### Theorem 5

*Let* ρ(*t*) *be real and of bounded variation on* [0, 1] *and the* ρ *be normalized, such that:*

(124) ρ(1)=0.

*Let:*

(125) $$A=\sqrt{\int_{0}^{1}\rho {\left(t\right)}^{2}dt}$$

*and let F*(*u*) *be a (real or complex) measurable function defined on* −∞ < *u* < ∞*. Then, a necessary and sufficient condition that:*

(126) $$F[\int_{0}^{1}x(t)d\rho (t)]$$

*be a Wiener measurable function of* *x*(•) *over* *C*_0_[0, 1] *is that:*

(127) $$e^{-\frac{u^{2}}{2}}F(Au)$$

*be of class L*_1_ *on* −∞ < *u* < ∞*. Moreover, if this condition is satisfied,*

(128) $$\int_{C_{0[0,1]}}F[\int_{0}^{1}x(t)d\rho (t)]d_{W}x=\frac{1}{\sqrt{2\pi }}\int_{-\infty }^{\infty }F(Au)e^{-\frac{u^{2}}{2}}du$$

We derive this equation below. Published results of a similar form may be found in [40], Theorem 29.7 (*n* = 1 case and assuming that ρ is normalized to unity). Other references are either Koval’chick [44] (page 106, Equation (13)) or Cameron and Martin [45] (page 393, Equation (6.3)). The same result is derived by an argument that will be familiar to many physicists in Paley, Wiener and Zygmund (see [46] Equation (2.11)), although the result derived there also assumes that the ρ is normalized to one. There is a difference of $\sqrt{2}$ between the result contained in Equation (128) and the older references [44,46], due to use of different definitions of the normal distribution used to define the Brownian motion on which the Wiener measure is based.

#### 7.1.1. Random Variables Derivation

We want to calculate:

(129) $$E_{W}(F(\varsigma \prime (t)))=\int_{C_{0[0,1]}}F(\varsigma \prime (t))d_{W}x$$

ς′(*t*) is a Gaussian random variable with zero mean, so ς′(*t*) ~ 𝒩 (0, σ^2^), where σ^2^ = *E_W_* (ς′(*t*)^2^). From this:

(130) $$E_{W}(F(\varsigma \prime (t)))=\int_{C_{0[0,1]}}F(\varsigma \prime (t))d_{W}x\;=\int_{-\infty }^{\infty }\frac{1}{\sigma \sqrt{2\pi }}e^{-\frac{u^{2}}{2\sigma^{2}}}F(u)du$$

### 7.2. The Second Wiener Integral

The second type of Wiener integral we need is:

(131) $$\int_{C_{0[0,1]}}d_{W}xF[\int_{0}^{1}x(t)d\rho_{1}(t),\ldots ,\int_{0}^{1}x(t)d\rho_{n}(t)]\;={\left(2\pi \right)}^{-n/2}\int_{-\infty }^{\infty }du_{1}\ldots \int_{-\infty }^{\infty }du_{n}F(u_{1},\ldots ,u_{n})e^{-\sum_{k=1}^{n}\frac{u_{k}^{2}}{2}}$$

where ρ_*i*_(1) = 0 for each index *i* and the ρ_*i*_ are orthonormal.

Similar versions of this integral appear in many sources, for instance in Koval’chick [44] (page 107, Equation (14)), which contains (after transcription into the modern conventions):

(132) $$\int_{C_{0}[0,1]}d_{W}xF[\int_{0}^{1}\rho_{1}(t)dx(t),\ldots ,\int_{0}^{1}\rho_{n}(t)dx(t)]\;={\left(2\pi \right)}^{-n/2}\int_{-\infty }^{\infty }du_{1}\ldots \int_{-\infty }^{\infty }du_{n}F(u_{1},\ldots ,u_{n})e^{-\sum_{k=1}^{n}\frac{u_{k}^{2}}{2}}$$

This form appears to be derivable from Equation (131) using integration by parts. Specifically,

(133) $$\int_{C_{0}[0,1]}d_{W}xF[\int_{0}^{1}\rho_{1}(t)dx(t),\ldots ,\int_{0}^{1}\rho_{n}(t)dx(t)]\;={\left(2\pi \right)}^{-n/2}\int_{-\infty }^{\infty }du_{1}\ldots \int_{-\infty }^{\infty }du_{n}F(u_{1},\ldots ,u_{n})e^{-\sum_{k=1}^{n}\frac{u_{k}^{2}}{2}}$$

We would formally transform this into the form of Equation (131) by the following steps:

First observe that the integral in Equation (133) is equal to:

(134) $$\int_{C_{0}[0,1]}d_{W}xF[-\int_{0}^{1}\rho_{1}(t)dx(t),\ldots ,-\int_{0}^{1}\rho_{n}(t)dx(t)]\;={\left(2\pi \right)}^{-n/2}\int_{-\infty }^{\infty }du_{1}\ldots \int_{-\infty }^{\infty }du_{n}F(-u_{1},\ldots ,-u_{n})e^{-\sum_{k=1}^{n}\frac{{\left(-u_{k}\right)}^{2}}{2}}\;={\left(2\pi \right)}^{-n/2}\int_{-\infty }^{\infty }du_{1}\ldots \int_{-\infty }^{\infty }du_{n}F(u_{1},\ldots ,u_{n})e^{-\sum_{k=1}^{n}\frac{u_{k}^{2}}{2}}$$

Next, use the facts that we have assumed that ρ_*i*_(1) = 0 and, additionally, that the Brownian paths are normalized according to *x*(0) = 0, so that (classical) integration by parts of the integrals in the first line of Equation (134) would yield:

(135) $$\int_{C_{0}[0,1]}d_{W}xF[\int_{0}^{1}x(t)d\rho_{1}(t),\ldots ,\int_{0}^{1}x(t)d\rho_{n}(t)]\;={\left(2\pi \right)}^{-n/2}\int_{-\infty }^{\infty }du_{1}\ldots \int_{-\infty }^{\infty }du_{n}F(u_{1},\ldots ,u_{n})e^{-\sum_{k=1}^{n}\frac{u_{k}^{2}}{2}}$$

Although this “derivation” shows that the two forms are equivalent, as desired, it overlooks the fact that the integrals in Equation (132) cannot be classical integrals. In fact, Wiener [47] (p. 68) states that integrals of the form:

(136) $$\int_{0}^{1}\rho_{i}(t)dx(t)$$

are actually Itō integrals. The correct integration-by-parts formula in this case is:

(137) $$X_{t}Y_{t}=X_{0}Y_{0}+\int_{0}^{t}X_{s-}dY_{s}+\int_{0}^{t}Y_{s-}dX_{s}+{\left[X,Y\right]}_{t}$$

where [*X, Y*]_*t*_ is the quadratic covariation process with:

(138) $${\left[X,Y\right]}_{t}≔\underset{\|P\|\to 0}{\text{lim}}\sum_{k=1}^{n}|X_{t_{k}}-X_{t_{k-1}}||Y_{t_{k}}-Y_{t_{k-1}}|$$

where *P* ranges over partitions of the interval [0, *t*] and the norm, ‖*P*‖, of the partition, *t*_0_ < ⋯ < *t_n_*, is the mesh, *i.e.*, max{|*t_i_* − *t*_*i*−1_| : *i* = 1, …, *n*}.

However, in the case where *Y* is of bounded variation:

(139) $${\left[X,Y\right]}_{t}\leq \text{max}\{|X_{t_{k}}-X_{T_{k-1}}|,k=1,\ldots ,n\}\underset{\|P\|\to 0}{\text{lim}}\sum_{k=1}^{n}|Y_{t_{k}}-Y_{T_{k-1}}|\;\leq \underset{\|P\|\to 0}{\text{lim}}\text{max}\{|X_{t_{k}}-X_{t_{k-1}}|,k=1,\ldots ,n\}V_{0}^{t}[Y]\;=0$$

so that Equation (137) reduces to the classical integration by parts formula. Thus, the derivation above is (accidentally) correct.

The number of sources containing detailed derivations of these equations in English appears to be limited. The only source we have been able to locate is Paley, Wiener and Zygmund, which is completely self-contained and contains the equivalent of our Equation (131) (see Equation (2.14) in [46]), although the result derived there assumes that the measure is normalized to one and uses a slightly different notation for the Brownian paths.

We need to compute Wiener integrals like those on the left-hand side of Equation (131) in the case where the ρ_*k*_(*t*) are not orthonormal. Moreover, we only need to consider the special form:

(140) $$\int_{c_{0}[0,1]}F_{1}[\int_{0}^{1}x(t)d\rho_{1}(t)]F_{2}[\int_{0}^{1}x(t)d\rho_{2}(t)]d_{W}x$$

To apply Equation (131), we use the Gram–Schmidt process to obtain an orthonormal family ν_1_(*t*), ν_2_(*t*) from the original ρ_1_(*t*), ρ_2_(*t*). Using the short-hand notation:

(141) $$\langle f(t),g(t)\rangle =\int_{0}^{1}f(t)g(t)dt$$

The Gram–Schmidt process is:

(142) $$\nu_{1}(t)=\frac{\rho_{1}(t)}{\sqrt{\langle \rho_{1},\rho_{1}\rangle }}=\frac{\rho_{1}(t)}{N_{1}}$$

$$\nu_{2}(t)=\frac{\rho_{2}(t)-\nu_{1}(t)\langle \rho_{2},\nu_{1}\rangle }{\sqrt{\langle \rho_{2}-\nu_{1}\langle \rho_{2},\nu_{1}\rangle ,\rho_{2}-\nu_{1}\langle \rho_{2},\nu_{1}\rangle \rangle }}\;=\frac{1}{N_{2}}\rho_{2}(t)-\frac{\langle \rho_{2},\nu_{1}\rangle }{N_{2}}\frac{1}{N_{1}}\rho_{1}(t)$$

where:

(143) $$N_{1}≔\sqrt{\langle \rho_{1},\rho_{1}\rangle }=\|\rho_{1}\|$$

$$N_{2}≔\sqrt{\langle \rho_{2}-\nu_{1}\langle \rho_{2},\nu_{1}\rangle ,\rho_{2}-\nu_{1}\langle \rho_{2},\nu_{1}\rangle \rangle }\;=\sqrt{\langle \rho_{2},\rho_{2}\rangle -2\langle \rho_{2},\nu_{1}\rangle \langle \rho_{2}\nu_{1}\rangle +{\langle \rho_{2},\nu_{1}\rangle }^{2}\langle \nu_{1},\nu_{1}\rangle }\;=\sqrt{\langle \rho_{2},\rho_{2}\rangle -2{\langle \rho_{2},\nu_{1}\rangle }^{2}+{\langle \rho_{2},\nu_{1}\rangle }^{2}}\;=\sqrt{\langle \rho_{2},\rho_{2}\rangle -\frac{1}{N_{1}^{2}}{\langle \rho_{1},\rho_{2}\rangle }^{2}}$$

which is expressed in matrix form:

(144) $$\left[\begin{array}{c} \nu_{1}(t)\\ \nu_{2}(t) \end{array}]=[\begin{array}{cc} \frac{1}{N_{1}} & 0\\ \frac{-\langle \rho_{1},\rho_{2}\rangle }{N_{1}N_{2}} & \frac{1}{N_{2}} \end{array}][\begin{array}{c} \rho_{1}(t)\\ \rho_{2}(t) \end{array}\right]$$

or:

(145) $$\left[\begin{array}{c} \rho_{1}(t)\\ \rho_{2}(t) \end{array}]=[\begin{array}{cc} N_{1} & 0\\ \langle \rho_{1},\rho_{2}\rangle & N_{2} \end{array}][\begin{array}{c} \nu_{1}(t)\\ \nu_{2}(t) \end{array}\right]$$

This permits us to rewrite Equation (140) as:

$$\int_{c_{0}[0,1]}F_{1}[N_{1}\int_{0}^{1}x(t)d\nu_{1}(t)]F_{2}[\langle \rho_{1},\rho_{2}\rangle \int_{0}^{1}x(t)d\nu_{1}(t)+N_{2}\int_{0}^{1}x(t)d\nu_{2}(t)]d_{W}x$$

to which (since the ν_*k*_(1) = 0, *k* = 1, 2) we may now apply Equation (131) to obtain:

(146) $$\int_{c_{0}[0,1]}F_{1}[N_{1}\int_{0}^{1}x(t)d\nu_{1}(t)]\times F_{2}[\langle \rho_{1},\rho_{2}\rangle \int_{0}^{1}x(t)d\nu_{1}(t)+N_{2}\int_{0}^{1}x(t)d\nu_{2}(t)]d_{W}x\;=\frac{1}{2\pi }\int_{-\infty }^{\infty }du_{1}\int_{-\infty }^{\infty }du_{2}e^{-\frac{u_{1}^{2}}{2}-\frac{u_{2}^{2}}{2}}F_{1}[N_{1}u_{1}]F_{2}[\langle \rho_{1},\rho_{2}\rangle u_{1}+N_{2}u_{2}]\;=\frac{1}{2\pi }\int_{-\infty }^{\infty }du_{1}\int_{-\infty }^{\infty }du_{2}e^{-\frac{u_{1}^{2}}{2}-\frac{u_{2}^{2}}{2}}F_{1}[\|\rho_{1}\|u_{1}]F_{2}[\langle \rho_{1},\rho_{2}\rangle u_{1}+N_{2}u_{2}]$$

## Supplementary Material

Appendix
