# Supplementary material for: Entropy vs. Energy Waveform Processing: A Comparison Based on the Heat Equation
Source: Entropy (Basel). Author manuscript; Available in PMC 2016 Apr 20. (PMC4838411; doi:10.3390/e17063518)
Supplement: Appendix [file NIHMS745315-supplement-Appendix.pdf]

7. A Short List of Wiener Integrals

7.1. The First Wiener Integral

The first type of Wiener integral described by the following

Theorem 3. *Let $\rho(t)$ be real and of bounded variation on $[0,1]$ and the ρ be normalized such that*

$$\rho(1) = 0. \tag{124}$$

Let

$$A = \sqrt{\int_0^1 \rho(t)^2 dt} \tag{125}$$

and let $F(u)$ be a (real or complex) measurable function defined on $-\infty < u < \infty$. Then a necessary and sufficient condition that

$$F\left[\int_0^1 x(t) d\rho(t)\right] \tag{126}$$

be a Wiener measurable function of $x(\bullet)$ over $C_0[0,1]$ is that

$$e^{-\frac{u^2}{2}} F(Au) \tag{127}$$

be of class L_1 on $-\infty < u < \infty$. Moreover, if this condition is satisfied,

$$\int_{C_0[0,1]} F\left[\int_0^1 x(t) d\rho(t)\right] d_W x = \frac{1}{\sqrt{2\pi}} \int_{-\infty}^{\infty} F(Au) e^{-\frac{u^2}{2}} du., \tag{128}$$

We derive this equation below. Published results of similar form may be found in [37], Theorem 29.7 ($n = 1$ case and assuming that ρ is normalized to unity). Other references are either Koval'chick reference [41] page 106, Eq. (13), or Cameron and Martin [42], page 393 of , Eq. (6.3). The same result is derived by an argument that will be familiar to many physicists in Paley, Wiener and Zygmund (see [43] Eq. (2.11)), although the result derived there also assumes that the ρ is normalized to one. There is a difference of $\sqrt{2}$ between the result contained in Eq. (128) and the older references ([41,43]), due to use of different definitions of the normal distribution used to define the Brownian motion on which the Wiener measure is based.

7.1.1. Random Variables Derivation

We want to calculate

$$E_W\left(F\left(\varsigma'(t)\right)\right) = \int_{C_0[0,1]} F\left(\varsigma'(t)\right) d_W x \tag{129}$$

$\varsigma'(t)$ is a Gaussian random variable with zero mean so $\varsigma'(t) \sim \mathcal{N}(0,\sigma^2)$,where $\sigma^2 = E_W\left(\varsigma'(t)^2\right)$. From this

$$\begin{aligned} E_W\left(F\left(\varsigma'(t)\right)\right) &= \int_{C_0[0,1]} F\left(\varsigma'(t)\right) d_W x, \\ &= \int_{-\infty}^{\infty} \frac{1}{\sigma\sqrt{2\pi}} e^{-\frac{u^2}{2\sigma^2}} F(u) du. \end{aligned} \tag{130}$$

7.2. The Second Wiener Integral

The second type of Wiener integral we need is

$$\int_{C_0[0,1]} d_W x\, F\left[\int_0^1 x(t)d\rho_1(t), \ldots, \int_0^1 x(t)d\rho_n(t)\right] \tag{131}$$
$$= (2\pi)^{-n/2} \int_{-\infty}^{\infty} du_1 \ldots \int_{-\infty}^{\infty} du_n F(u_1, \ldots, u_n) e^{-\sum_{k=1}^{n} \frac{u_k^2}{2}},$$

where $\rho_i(1) = 0$ for each index i and the ρ_i are orthonormal.

Similar versions of this integral appear in many sources, for instance in Koval'chick [41] page 107, Eq. (14), which contains (after transcription into the modern conventions)

$$\int_{C_0[0,1]} d_W x\, F\left[\int_0^1 \rho_1(t)dx(t), \ldots, \int_0^1 \rho_n(t)dx(t)\right] \tag{132}$$
$$= (2\pi)^{-n/2} \int_{-\infty}^{\infty} du_1 \ldots \int_{-\infty}^{\infty} du_n F(u_1, \ldots, u_n) e^{-\sum_{k=1}^{n} \frac{u_k^2}{2}}.$$

This form appears to be derivable from Eq. (131) using integration by parts. Specifically,

$$\int_{C_0[0,1]} d_W x\, F\left[\int_0^1 \rho_1(t)dx(t), \ldots, \int_0^1 \rho_n(t)dx(t)\right] \tag{133}$$
$$= (2\pi)^{-n/2} \int_{-\infty}^{\infty} du_1 \ldots \int_{-\infty}^{\infty} du_n F(u_1, \ldots, u_n) e^{-\sum_{k=1}^{n} \frac{u_k^2}{2}},$$

We would formally transform this into the form of Eq. (131) by the following steps:

First observe that the integral in Eq. (133) is equal to

$$\int_{C_0[0,1]} d_W x\, F\left[-\int_0^1 \rho_1(t)dx(t), \ldots, -\int_0^1 \rho_n(t)dx(t)\right] \tag{134}$$
$$= (2\pi)^{-n/2} \int_{-\infty}^{\infty} du_1 \ldots \int_{-\infty}^{\infty} du_n F(-u_1, \ldots, -u_n) e^{-\sum_{k=1}^{n} \frac{(-u_k)^2}{2}}$$
$$= (2\pi)^{-n/2} \int_{-\infty}^{\infty} du_1 \ldots \int_{-\infty}^{\infty} du_n F(u_1, \ldots, u_n) e^{-\sum_{k=1}^{n} \frac{u_k^2}{2}},$$

Next, use the facts that we have assumed that $\rho_i(1) = 0$, and additionally that the Brownian paths are normalized according to $x(0) = 0$, so that (classical) integration-by-parts of the integrals in the first line of Eq. (134) would yield

$$\int_{C_0[0,1]} d_W x\, F\left[\int_0^1 x(t)d\rho_1(t), \ldots, \int_0^1 x(t)d\rho_n(t)\right] \tag{135}$$
$$= (2\pi)^{-n/2} \int_{-\infty}^{\infty} du_1 \ldots \int_{-\infty}^{\infty} du_n F(u_1, \ldots, u_n) e^{-\sum_{k=1}^{n} \frac{u_k^2}{2}}.$$

Although this "derivation" shows that the two forms are equivalent as desired, it overlooks the fact that the integrals in Eq.(132) cannot be classical integrals. In fact, Wiener ([44], pg. 68) states that integrals of the form

$$\int_0^1 \rho_i(t)dx(t), \tag{136}$$

are actually Itō integrals. The correct integration-by-parts formula in this case is

$$X_t Y_t = X_0 Y_0 + \int_0^t X_{s-} dY_s + \int_0^t Y_{s-} dX_s + [X, Y]_t, \tag{137}$$

where $[X, Y]_t$ is the quadratic covariation process with

$$[X, Y]_t := \lim_{\|P\| \to 0} \sum_{k=1}^{n} |X_{t_k} - X_{t_{k-1}}||Y_{t_k} - Y_{t_{k-1}}|, \tag{138}$$

where P ranges over partitions of the interval $[0, t]$ and the norm, $\|P\|$, of the partition, $t_0 < \cdots < t_n$, is the mesh, *i.e.*, $\max\{|t_i - t_{i-1}| : i = 1, \ldots, n\}$.

However, in the case where Y is of bounded variation

$$\begin{aligned} [X, Y]_t \leq & \max\{|X_{t_k} - X_{T_{k-1}}|, k = 1, \ldots, n\} \lim_{\|P\| \to 0} \sum_{k=1}^{n} |Y_{t_k} - Y_{T_{k-1}}|, \\ \leq & \lim_{\|P\| \to 0} \max\{|X_{t_k} - X_{t_{k-1}}|, k = 1, \ldots, n\} V_0^t[Y], \\ = & 0, \end{aligned} \tag{139}$$

so that Eq. (137) reduces to the classical integration-by-parts formula. Thus, the derivation above is (accidentally) correct.

The number of sources containing detailed derivations of these equations in English appears to be limited. The only source we have been able to locate is Paley, Wiener and Zygmund which is completely self-contained and contains the equivalent of our Eq. (131) (see [43] Eq. (2.14)), although the result derived there assumes that the measure is normalized to one and uses a slightly different notation for the Brownian paths.

We need to compute Wiener integrals like those on the left-hand side of Eq. (131) in the case where the $\rho_k(t)$ are not orthonormal. Moreover, we only need to consider the special form

$$\int_{c_0[0,1]} F_1 \left[\int_0^1 x(t) d\rho_1(t) \right] F_2 \left[\int_0^1 x(t) d\rho_2(t) \right] d_W x. \tag{140}$$

To apply Eq. (131) we use the Gram-Schmidt process to obtain an orthonormal family $\nu_1(t), \nu_2(t)$ from the original $\rho_1(t), \rho_2(t)$. Using the short-hand notation

$$\langle f(t), g(t) \rangle = \int_0^1 f(t) g(t) dt, \tag{141}$$

The Gram-Schmidt process is

$$\begin{aligned} \nu_1(t) = & \frac{\rho_1(t)}{\sqrt{\langle \rho_1, \rho_1 \rangle}} = \frac{\rho_1(t)}{N_1}, \\ \nu_2(t) = & \frac{\rho_2(t) - \nu_1(t)\langle \rho_2, \nu_1 \rangle}{\sqrt{\langle \rho_2 - \nu_1 \langle \rho_2, \nu_1 \rangle, \rho_2 - \nu_1 \langle \rho_2, \nu_1 \rangle \rangle}} \\ = & \frac{1}{N_2} \rho_2(t) - \frac{\langle \rho_2, \nu_1 \rangle}{N_2} \frac{1}{N_1} \rho_1(t), \end{aligned} \tag{142}$$

where

$$\begin{aligned} N_1 :=& \sqrt{\langle \rho_1, \rho_1 \rangle} = \|\rho_1\|, \\ N_2 :=& \sqrt{\langle \rho_2 - \nu_1 \langle \rho_2, \nu_1 \rangle, \rho_2 - \nu_1 \langle \rho_2, \nu_1 \rangle \rangle}, \\ =& \sqrt{\langle \rho_2, \rho_2 \rangle - 2\langle \rho_2, \nu_1 \rangle \langle \rho_2, \nu_1 \rangle + \langle \rho_2, \nu_1 \rangle^2 \langle \nu_1, \nu_1 \rangle}, \\ =& \sqrt{\langle \rho_2, \rho_2 \rangle - 2\langle \rho_2, \nu_1 \rangle^2 + \langle \rho_2, \nu_1 \rangle^2}, \\ =& \sqrt{\langle \rho_2, \rho_2 \rangle - \frac{1}{N_1^2} \langle \rho_1, \rho_2 \rangle^2}, \end{aligned} \tag{143}$$

which is expressed in matrix form

$$\begin{bmatrix} \nu_1(t) \\ \\ \nu_2(t) \end{bmatrix} = \begin{bmatrix} \frac{1}{N_1} & 0 \\ \\ \frac{-\langle \rho_1, \rho_2 \rangle}{N_1 N_2} & \frac{1}{N_2} \end{bmatrix} \begin{bmatrix} \rho_1(t) \\ \\ \rho_2(t) \end{bmatrix} \tag{144}$$

or

$$\begin{bmatrix} \rho_1(t) \\ \\ \rho_2(t) \end{bmatrix} = \begin{bmatrix} N_1 & 0 \\ \\ \langle \rho_1, \rho_2 \rangle & N_2 \end{bmatrix} \begin{bmatrix} \nu_1(t) \\ \\ \nu_2(t) \end{bmatrix}. \tag{145}$$

This permits us rewrite Eq. (140) as

$$\int_{c_0[0,1]} F_1 \left[N_1 \int_0^1 x(t) d\nu_1(t) \right] F_2 \left[\langle \rho_1, \rho_2 \rangle \int_0^1 x(t) d\nu_1(t) + N_2 \int_0^1 x(t) d\nu_2(t) \right] d_W x$$

To which (since the $\nu_k(1) = 0$, $k = 1, 2$) we may now apply Eq. (131) to obtain

$$\begin{aligned} \int_{c_0[0,1]} & F_1 \left[N_1 \int_0^1 x(t) d\nu_1(t) \right] \\ & \times F_2 \left[\langle \rho_1, \rho_2 \rangle \int_0^1 x(t) d\nu_1(t) + N_2 \int_0^1 x(t) d\nu_2(t) \right] d_W x \\ =& \frac{1}{2\pi} \int_{-\infty}^{\infty} du_1 \int_{-\infty}^{\infty} du_2 e^{-\frac{u_1^2}{2} - \frac{u_2^2}{2}} F_1 \left[N_1 u_1 \right] F_2 \left[\langle \rho_1, \rho_2 \rangle u_1 + N_2 u_2 \right], \\ =& \frac{1}{2\pi} \int_{-\infty}^{\infty} du_1 \int_{-\infty}^{\infty} du_2 e^{-\frac{u_1^2}{2} - \frac{u_2^2}{2}} F_1 \left[\|\rho_1\| u_1 \right] F_2 \left[\langle \rho_1, \rho_2 \rangle u_1 + N_2 u_2 \right]. \end{aligned} \tag{146}$$
